# Mesocarnivore landscape use along a gradient of urban, rural, and forest cover

**DOI:** 10.7717/peerj.11083

**Published:** 2021-04-06

**Authors:** Jordan T. Rodriguez, Damon B. Lesmeister, Taal Levi

**Affiliations:** 1 Pacific Northwest Research Station, USDA Forest Service, Corvallis, OR, United States of America; 2 Department of Fisheries and Wildlife, Oregon State University, Corvallis, OR, United States of America

**Keywords:** Bobcat (*Lynx rufus*), Coyote (*Canis latrans*), Gray fox (*Urocyon cinereoargenteus*), Raccoon (*Procyon lotor*), Striped skunk (*Mephitis mephitis*), Virgina opossums (*Didelphis virginiana*), Human disturbance, Activity, Mammalian carnivores, Landscape use

## Abstract

Mesocarnivores fill a vital role in ecosystems through effects on community health and structure. Anthropogenic-altered landscapes can benefit some species and adversely affect others. For some carnivores, prey availability increases with urbanization, but landscape use can be complicated by interactions among carnivores as well as differing human tolerance of some species. We used camera traps to survey along a gradient of urban, rural, and forest cover to quantify how carnivore landscape use varies among guild members and determine if a species was a human exploiter, adapter, or avoider. Our study was conducted in and around Corvallis, Oregon from April 2018 to February 2019 (11,914 trap nights) using 47 camera trap locations on a gradient from urban to rural. Our focal species were bobcat (*Lynx rufus*), coyote (*Canis latrans*), gray fox (*Urocyon cinereoargenteus*), opossum (*Didelphis virginiana*), raccoon (*Procyon lotor*), and striped skunk (*Mephitis mephitis*). Raccoon and opossum were human exploiters with low use of forest cover and positive association with urban and rural developed areas likely due to human-derived resources as well as some refugia from larger predators. Coyote and gray fox were human adapters with high use of natural habitats while the effects of urbanization ranged from weak to indiscernible. Bobcat and striped skunk appeared to be human avoiders with negative relationship with urban cover and higher landscape use of forest cover. We conducted a diel temporal activity analysis and found mostly nocturnal activity within the guild, but more diurnal activity by larger-bodied predators compared to the smaller species. Although these species coexist as a community in human-dominated landscapes throughout much of North America, the effects of urbanization were not equal across species. Our results, especially for gray fox and striped skunk, are counter to research in other regions, suggesting that mesopredator use of urbanized landscapes can vary depending on the environmental conditions of the study area and management actions are likely to be most effective when decisions are based on locally derived data.

## Introduction

Habitat loss and fragmentation is one of the greatest threats to global biodiversity (Butchart et al., 2010; Seto, Guneralp & Hutyra, 2012). Urbanization and agricultural development are among the chief drivers of this phenomenon (Imhoff et al., 2004; Aronson et al., 2014), but a suite of human commensal species also benefit from anthropogenic landscapes (Baker & Harris, 2007; Soulsbury & White, 2016). This dichotomy is particularly true for mammalian carnivores, some of which are frequently extirpated while others reach their highest population densities in anthropogenic landscapes. Many mammalian carnivores benefit either from human-derived resources or release from predation in anthropogenic landscapes (Pickett et al., 2001; Prange, Gehrt & Wiggers, 2003; Gaston et al., 2005). The variable species response to human disturbance can complicate our understanding of community structure and landscape use of the urban-rural-forest matrix (Lesmeister et al., 2015; Moss, Alldredge & Pauli, 2016).

Species most susceptible to anthropogenic disturbance include those with large home ranges, low population densities, or low fecundity (Pimm et al., 2014). Apex predators, which frequently share these traits, have declined globally in fragmented and urbanized systems (Crooks & Soulé, 1999; Hollings et al., 2016) while many mesopredators with faster life histories, smaller home ranges, and higher fecundities have increased their distribution and abundance (Prugh et al., 2009; Santini et al., 2019). The ability of species to persist in anthropogenic environments is also a product of human attitudes that lead to the persecution of species, such as apex predators, that are thought to be dangerous or damaging to human interests (Kansky & Knight, 2014).

The local extirpation of apex predators can release herbivorous prey species and mesopredators (Ritchie & Johnson, 2009; Prugh et al., 2009). Although mesopredators can benefit from anthropogenic subsidies and predation release (Pickett et al., 2001; Prange, Gehrt & Wiggers, 2003; Gaston et al., 2005), they incur substantial risk in human-dominated landscapes (Woods, Mcdonald & Harris, 2003; Baker et al., 2004; Blanchoud, Farrugia & Mouchel, 2004). Risks in urban landscapes include increased vehicle traffic, lethal removals, and domesticated pets (Lowry, Lill & Wong, 2013; Soulsbury & White, 2016). Animals living within urban environments may consequently alter daily activity patterns compared to their rural or forested counterparts due to human activity during diurnal hours (Grinder & Krausman, 2001; George & Crooks, 2006). Individual species within diverse carnivore communities, such as bobcat *(Lynx rufus*), coyote (*Canis latrans*), gray fox (*Urocyon cinereoargenteus*), opossum (*Didelphis virginiana*), raccoon (*Procyon lotor*), and striped skunk (*Mephitis mephitis*), may partition time to maximize resource gain and reduce energy consumption by avoiding human areas during daylight hours (Schuette et al., 2013; Wang, Allen & Wilmers, 2015).

Here we combined camera traps across a landscape gradient of urban, rural, and forested land cover with occupancy models to quantify effects of the multi-scaled landscape features on landscape use by mesopredators, including bobcat, coyote, gray fox, opossum, raccoon, and striped skunk, while accounting for imperfect detection (Lindstedt, Miller & Buskirk, 1986; Crooks, 2002). Within this guild of mesopredators, we expected some species would be human commensals while others would have less affinity towards human development. We expected to classify species as human avoiders, human adapters, or human exploiters (Blair, 1996; Riggio et al., 2018). Species that were positively associated with natural habitats and negatively associated with human developments were classified as human avoiders. Species that were negatively associated with natural habitats and positively associated with urbanization were classified as human exploiters. Species that did not show strong preference for either natural or human habitats were classified as human adapters. We assessed the response of each species to land cover type across three spatial scales (100 m, 500 m, and 1000 m) to determine the most important scale of response. Spatial scale is important when predicting species use (e.g., Gompper et al., 2016), and we expected that smaller species would respond to differences in cover types at a finer scale (Jetz et al., 2004).

Based on previous research, we predicted that the larger-bodied mesopredators (coyote and particularly bobcat) would be human avoiders that were negatively associated with anthropogenic disturbance (Atwood, Weeks & Gehring, 2004; Clare, Anderson & Macfarland, 2015; Lesmeister et al., 2015; Flores-Morales et al., 2019). In many regions raccoon and opossum have been found associated with urban habitats that provide human-subsidized resources and reduced risk from natural predators, so we expected them to be human exploiters in our study area (Prange, Gehrt & Wiggers, 2003; Bateman & Fleming, 2012). We predicted that gray fox and striped skunk would be human adapters with limited affinity for, or avoidance, of anthropogenic landscapes. Striped skunks are known to use human structures as denning sites (Theimer et al., 2017) and are commonly associated with rural human settlements (Lesmeister et al., 2015). Gray fox is commonly described as a species that associates with human development (Riley, 2006; Lesmeister et al., 2015), but that association can vary greatly between study areas and may relate more to site specific factors such as interference competition for prey or the prevalence of free-ranging dogs (Morin et al., 2018). We anticipated modest responses to forests, grasslands, and wetlands among species because all are capable of exploiting a variety of vegetation types, with the exception of raccoon, which we expected would be substantially associated with water cover (Henner et al., 2004).

## Materials and Methods

### Subjects

We classified our six target species (bobcat, coyote, gray fox, opossum, raccoon, and striped skunk) into three categories of human relationship (exploiter, adapter, and avoider) based on their occupancy use related to landscape features. From Blair (1996), exploiters will reach greater densities in sites with urban development and avoiders will be sensitive to features such as housing development and roads while preferring less disturbed natural areas like forests and grassland. Adapters will not fit neatly into the previous two classifications, but would be expected to benefit from additional human supplied resources like shelter and food (Blair, 1996; Riggio et al., 2018). We classified species as human adapters if there was no indication of a strong relationship with urban features, positive or negative. We expected these species to perhaps show weak relationships with both natural and anthropogenic landscape features. Landscape features we considered associated with human development were urban cover, distance to structure, and paved and unpaved roads. Forest cover, grassland cover, water cover, and distance to water were considered natural features when classifying our species.

### Study area

**Figure 1:**
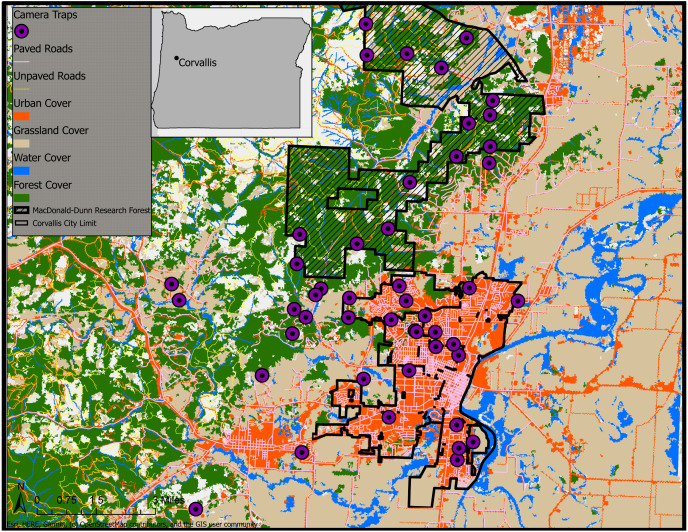
Land cover classifications (NLCD 2011) and camera trap survey locations in Corvallis, Benton County, Oregon, USA, April 2018–February 2019. Map data ©2019 OpenStreetMap contributors.

We conducted a camera trap survey across urban, rural, and forested cover types in and around Corvallis, Oregon, USA (Fig. 1). We considered urban camera stations those that were within the city limits of Corvallis. Camera traps defined as rural were placed at sites outside city limits but remained on lands with human dwellings with the appropriate permission from landowners. The forest cover type was restricted to the MacDonald-Dunn Research Forest that bordered Corvallis to the northwest and contained no human dwellings.

According to the U.S. Census Bureau (2018), Corvallis had a population of 58,641 and covers 54.67 km^2^. The population density was 1,547.2/km^2^, with an average housing unit density of 665.4/km^2^ (US Census Bureau, 2018). Corvallis, and the rest of the Willamette Valley in Oregon, is located in the dry-summer temperate climate zone, or cool-summer Mediterranean (Kottek et al., 2006). The climate was characterized by year-round mild temperatures with warm, dry summers and cool, wet winters. Average yearly precipitation in Corvallis was 110.9 cm and an average high temperature of 63.4°F (17.4 °C) and an average low temperature of 41.9°F (5.5 °C). The average high and low temperatures in January were 47.0°F (8.3 °C) and 33.6°F (0.9 °C) respectively, and average precipitation of 16.4 cm (US Climate Data, 1981-2010). The average high and low temperatures in July were 81.2°F (27.3 °C) and 51.8°F (11.0 °C) respectively, and average precipitation of 1.4 cm (US Climate Data, 1981-2010).

The McDonald-Dunn Research Forest was managed by Oregon State University and open to public recreation. The forest was over 4450 ha and rested in the eastern foothills of Oregon’s coastal mountain range, extending into the western Willamette Valley (Edson & Wing, 2011). Historically, much of the lowland MacDonald-Dunn Research Forest was dominated by oak savannas, but due to disturbance and other environmental conditions the forest was dominated by an overstory of Douglas fir (*Pseudotsuga menziesii*) (Fletcher et al., 2005). Other common native species include big-leaf maple (*Acer macrophyllum*), vine maple (*A. circinatum*), Oregon grape (*Mahonia aquifolium*), and western sword fern (*Polystichum munitum*). The McDonald-Dunn Research Forest was subject to four different forest management types (even-aged, two-storied, uneven-aged, and reserved old-growth stands) (OSU Research Forests). The forest was not open to public vehicle access but does receive over 175,000 recreation visits per year.

### Site selection and data collection

We deployed 47 non-baited camera traps (Bushnell, Overland Park, KS, Trophy Cam HD) at urban, rural, and forest sites extending out from Corvallis, Oregon during April–June 2018. We revisited sites every four to six weeks to collect image data and service camera traps for a total of 41 weeks. We placed camera traps on private landowner properties as well as the MacDonald-Dunn Research Forest. We selected locations along the urban to rural gradient to sample as evenly as possible across the densest areas of Corvallis to the lesser populated rural dwellings surrounding the city. We attempted to select locations for camera traps that were no closer than 500 m to avoid redundancy in animal detections and land cover sampled. However, there were instances of camera trap locations that fell within this 500 m buffer due to our site selection process relying upon voluntary involvement from private landowners. Spacing of sampling sites was easier in the MacDonald-Dunn Research Forest since we were not restricted by property ownership and permissions from multiple landowners. Our goal for site selection was to get an even distribution of cameras between our three zones: urban, rural, and forest. We deployed 18 camera traps in urban areas, 14 in rural areas, and 15 in the MacDonald-Dunn Research Forest. A variety of cover types were selected for camera trap placements: mature forest, young forest, thinned stands, clear cuts, oak savannas, and riparian zones.

We placed camera traps at the bases of trees, fence posts, or other suitable structures approximately 50 cm off the ground. We set the cameras to record the time, date, temperature, and lunar phase of each image. We adapted our camera settings based on previous studies such as Wang, Allen & Wilmers (2015). We set the cameras to take three images in rapid succession for each detection event to improve the chances of species identification. A quiet period of one minute was set after a detection event to reduce duplicate detections of the same individual detection event and conserve battery life. We noted that previous findings from Ridout & Linkie (2009) found that a 30 min delay was a useful time delay in discerning detections of the same species, however our analysis did not rely significantly on individual activity patterns. We identified and tagged images by species and used those detections to generate weekly encounter histories for each species at each site so that one or more detections weekly was coded as “1” and no detections as “0”.

### Survey and site variables

We summarized weekly temperature (TEMP) and precipitation (PRECIP) data that were collected from a nearby weather station (Corvallis, Oregon AgriMet Weather Station) to use in our detection probability models. We also used a binary factor to indicate that the camera was placed on an unpaved road (ONROAD; Table 1).

**Table 1:** Codes and covariate descriptions for detection and occupancy models for mesocarnivores in and near Corvallis, OR, April 2018-February 2019. For occupancy models, we measured variables in 3 buffer sizes (100 m, 500 m, and 1000 m) at each camera survey location. Also included are survey variables to inform our detection probability models.

Covariate	CODE	Description
precipitation	PRECIP	sum of weekly precipitation recorded from Corvallis weather station (AgriMet Station)
temperature	TEMP	average weekly temperature recorded from Corvallis weather station (AgriMet Station)
camera on road	ONROAD	whether or not a camera was placed on a road
distance to human structure	DTSTRUC	distance (m) to nearest human structure, measured with rangefinder if structure was visible; if not visible, measured with GIS
distance to surface water	DTWATER	distance (m) to nearest surface water measured with GIS
density of unpaved roads 100 m	USUMRD100 m	sum of unpaved road length in 100 m buffer around site
density of unpaved roads 500 m	USUMRD500 m	sum of unpaved road length in 500 m buffer around site
density of unpaved roads 1000 m	USUMRD1000 m	sum of unpaved road length in 1000 m buffer around site
density of paved roads 100 m	PSUMRD100 m	sum of paved road length in 100 m buffer around site
density of paved roads 500 m	PSUMRD500 m	sum of paved road length in 500 m buffer around site
density of paved roads 1000 m	PSUMRD1000 m	sum of paved road length in 1000 m buffer around site
forest cover 100 m	FC100 m	percent forest cover in a 100 m buffer around survey site measured with GIS
forest cover 500 m	FC500 m	percent forest cover in a 500 m buffer around survey site measured with GIS
forest cover 1000 m	FC1000 m	percent forest cover in a 1000 m buffer around survey site measured with GIS
urban cover 100 m	UC100 m	percent urban cover in a 100 m buffer around survey site measured with GIS
urban cover 500 m	UC500 m	percent urban cover in a 500 m buffer around survey site measured with GIS
urban cover 1000 m	UC1000 m	percent urban cover in a 1000 m buffer around survey site measured with GIS
grassland cover 100 m	GC100 m	percent grassland cover in a 100 m buffer around survey site measured with GIS
grassland cover 500 m	GC500 m	percent grassland cover in a 500 m buffer around survey site measured with GIS
grassland cover 1000 m	GC1000 m	percent grassland cover in a 1000 m buffer around survey site measured with GIS
water cover 100 m	WC100 m	percent water cover in a 100 m buffer around survey site measured with GIS
water cover 500 m	WC500 m	percent water cover in a 500 m buffer around survey site measured with GIS
water cover 1000 m	WC1000 m	percent water cover in a 1000 m buffer around survey site measured with GIS

We used program ArcGIS (ESRI, Redlands, CA) to generate 100 m, 500 m, and 1000 m buffers around each camera trap site. Using the 2011 National Land Cover Data (Multi-Resolution Land Characteristics Consortium), we summarized forest (deciduous, evergreen, and mixed forest), urban development (open space, low intensity, medium intensity, high intensity), grassland (hay/pasture and cultivated crops), and water (streams, woody wetland, emergent herbaceous wetland, surface water) land cover classes. Within each buffer, we created spatial variables by calculating the proportion of land cover classes of forest (FC100M, FC500M, FC1000M), urban (UC100M, UC500M, UC1000M), grassland (GC100M, GC500M, GC1000M), and water (WC100M, WC500M, WC1000M). We used data from the Oregon Department of Transportation (Oregon Spatial Data Library) to calculate the summed length of unpaved roads (USUMRD100M, USUMRD500M, USUMRD1000M) and paved roads (PSUMRD100M, PSUMRD500M, PSUMRD1000M). We used data from the Oregon Water Resources Department (Oregon Spatial Data Library) to determine the distance to surface water (DTWATER) from each camera trap. If a human structure was visible from the camera site, we calculated the distance (DTSTRUC) using a rangefinder (Table 1). If no structure was immediately visible, we used aerial imagery in ArcGIS to measure distance.

### Temporal activity analysis

The time and date of each image in our study was recorded. Images of the same species were not included in the temporal activity analysis if they were observed within 30 min of each other to reduce pseudoreplication (Ridout & Linkie, 2009; Lucherini et al., 2009; Wang, Allen & Wilmers, 2015). We reasoned that detections >30 min apart at unbaited cameras could be assumed independent events in activity analysis. Detection events with two or more individuals of the same species were counted as a single event. In the case of two different species in a single image, the event was recorded once for each species. We used the camtrapR 1.5.3 package (Niedballa et al., 2016) in RStudio 3.5.3 to create single-species diel activity kernel density estimation plots.

### Occupancy modeling

We used Program Presence 2.12.17 and RPresence 2.12.17 (Mackenzie et al., 2002; Hines, 2006; MacKenzie, Nichols & Royle, 2018) in a two-stage approach to fit and rank our single-species single-season occupancy models (MacKenzie, Nichols & Royle, 2018). First, we modeled our survey level covariates (TEMP, PRECIP, and ONROAD) against a null (.) detection model to determine which covariate was most supported for detection probability (*p*) (Appendix A). We used the most supported model structure based on The Akaike information criterion (Akaike, 1974) for small sample sizes (AIC_c_) values for detection probability in subsequent models that included covariates for occupancy (Ψ). We then fit univariate occupancy models and varied the value of the focal covariate based on three different buffers (100 m, 500 m, 1000 m). We carried forward the buffer scale with the most AIC_c_ weight for use in our multivariate models. We did not include covariates in the same multivariate model if their correlation coefficient had a magnitude >—.6—. In general, correlated covariates included covariates of the same cover type at different spatial scales. At most spatial scales, paved road density was negatively correlated with forest cover and positively correlated with urban cover. Unpaved road density was positively correlated with distance to structures. For each species, we compared the strength of multivariate models (including intercept only) and carried forward the models (and associated covariate beta coefficients) that collectively comprised >90% of model weight into our analysis (Table 2, Appendix B). We relied on model weight for final model selection rather than the ΔAIC_c_ ≤ 2 threshold because it has been found to be insufficient for within-stage model selection (Morin et al., 2020) and may exclude models with strong support.

## Results

Our 47 cameras were operational for 11,914 trap nights over the 41-week study period yielding 23,988 images of 14 identified vertebrate species. Columbian black-tailed deer (*Odocoileus hemionus columbianus*) were the most commonly detected species (*n* = 5371) at 42 sites. Raccoon was the most commonly detected carnivore (*n* = 1964) at 29 sites. There were 1680 detections of opossum at 31 sites, 471 detections of gray fox at 19 sites, 415 detections of striped skunk at 18 sites, 171 detections of coyote at 13 sites, and 134 detections of bobcat at 20 sites. We detected cougar (*Puma concolor*) 30 times at eight sites. Black bear (*Ursus americanus*) was detected once at a single site.

**Table 2:** Most supported occupancy models for bobcat, coyote, gray fox, opossum, raccoon, and striped skunk from 47 camera trap locations in and near Corvallis, OR. Here we include the top three models for each species. For all models, detection probability (p) was the most supported survey level model for each species. Null occupancy models were excluded if they were not included within the top 90% of model weight.

Model^a^	Δ AIC_c_^b^	*ω*^c^	K^d^	neg2ll^e^		*β*^f^	se^g^
**Bobcat (*Lynx rufus*)**							
Ψ(UC100 m+GC100 m), *p* (ONROAD)	0	0.3415	5	569.55	Ψ(UC100 m)	−1.080	0.430
					Ψ(GC100 m)	0.786	0.645
					p(ONROAD)	1.068	0.251
							
Ψ(UC100 m), *p* (ONROAD)	0.23	0.3047	4	572.29	Ψ(UC100 m)	−1.184	0.412
					p(ONROAD)	1.053	0.247
							
Ψ(GC100 m+FC1000 m), *p* (ONROAD)	2.21	0.1129	5	571.76	Ψ(GC100 m)	1.139	0.615
					Ψ(FC1000 m)	0.811	0.359
					p(ONROAD)	1.049	0.247
**Coyote (*Canis latrans*)**							
Ψ(USUMRD100 m+WC500 m), *p* (Precip)	0	0.140	5	844.12	Ψ(USUMRD100 m)	0.785	0.374
					Ψ(WC500 m)	0.729	0.394
					p(Precip)	−0.541	0.116
							
Ψ(WC500 m+FC100 m), *p* (Precip)	0.85	0.092	5	844.97	Ψ(WC500 m)	0.781	0.416
					Ψ(FC100 m)	0.740	0.370
					p(Precip)	−0.541	0.116
Ψ(USUMRD100 m+WC500 m+FC100 m), *p* (Precip)	1.75	0.059	6	843.23	Ψ(USUMRD100 m)	0.554	0.437
					Ψ(WC500 m)	0.832	0.423
					Ψ(FC100 m)	0.407	0.437
					p(Precip)	−0.541	0.116
**Gray Fox (*Urocyon cinereoargenteus*)**							
Ψ(GC1000 m), *p* (ONROAD)	0	0.426	4	757.62	Ψ(GC1000 m)	1.192	0.419
					p(ONROAD)	−1.558	0.269
Ψ(GC1000 m+PSUMRD500 m), *p* (ONROAD)	0.87	0.276	5	755.97	Ψ(GC1000 m)	1.087	0.420
					Ψ(PSUMRD500 m)	−0.470	0.380
					p(ONROAD)	−1.566	0.271
							
Ψ(GC1000 m+UC500 m), *p* (ONROAD)	2.5	0.122	5	757.61	Ψ(GC1000 m)	1.182	0.430
					Ψ(UC500 m)	−0.034	0.350
					p(ONROAD)	−1.558	0.269
**Opossum (*Didelphis virginiana)***							
Ψ(DTSTRUC), *p* (Temp)	0	0.1863	4	1459.36	Ψ(DTSTRUC)	−1.777	0.660
					p(Temp)	0.209	0.067
Ψ(DTSTRUC+UC100 m), *p* (Temp)	0.015	0.1848	5	1456.86	Ψ(DTSTRUC)	−1.204	0.617
					Ψ(UC100 m)	0.804	0.571
					p(Temp)	0.209	0.067
Ψ(USUMRD500 m), *p* (Temp)	0.467	0.1475	4	1459.83	Ψ(USUMRD500 m)	−1.322	0.406
					p(Temp)	0.209	0.067
**Raccoon (*Procyon lotor*)**							
Ψ(rDTSTRUC), *p* (ONROAD)	0	0.392	4	1414.47	Ψ(DTSTRUC)	−1.573	0.633
					p(ONROAD)	−2.595	0.376
Ψ(DTSTRUC+PSUMRD100 m), *p* (ONROAD)	2.05	0.1404	5	1414.02	Ψ(DTSTRUC)	−1.318	0.688
					Ψ(PSUMRD100 m)	0.311	0.469
					p(ONROAD)	−2.581	0.374
Ψ(DTSTRUC+UC500 m), *p* (ONROAD)	2.47	0.1139	5	1414.44	Ψ(DTSTRUC)	−1.494	0.734
					Ψ(UC500 m)	0.088	0.445
					p(ONROAD)	−2.591	0.376
**Skunk (*Mephitis mephitis*)**							
Ψ(FC500 m), *p* (ONROAD)	0	0.3583	4	708.01	Ψ(FC500 m)	0.875	0.337
					p(ONROAD)	−0.674	0.222
Ψ(PSUMRD100 m), *p* (ONROAD)	1.84	0.1429	4	709.85	Ψ(PSUMRD100 m)	−0.825	0.380
					p(ONROAD)	−0.673	0.221
Ψ(UC1000 m), *p* (ONROAD)	2.06	0.1281	4	710.07	Ψ(UC1000 m)	−0.824	0.393
					p(ONROAD)	−0.673	0.221

**Notes.**

a FC100 m, percent forest cover within 100 m radius; FC500 m, percent forest cover within 500 m radius; FC1000 m, percent forest cover within 1000 m radius; UC100 m, percent urban cover within 100 m radius; UC500 m, percent urban cover within 500 m radius; UC1000 m, percent urban cover within 1000 m radius; GC100 m, percent grassland cover within 100 m radius; GC500 m, percent grassland cover within 500 m radius; GC1000 m, percent grassland cover within 1000 m radius; WC100 m, percent water cover within 100 m radius; WC500 m, percent water cover within 500 m radius; WC1000 m, percent water cover within 1000 m radius; USUMRD100 m, summed length of unpaved roads in 100 m radius; USUMRD500 m, summed length of unpaved roads in 500 m radius; USUMRD1000 m, summed length of unpaved roads in 1000 m radius; PSUMRD100 m, summed length of paved roads in 100 m radius; PSUMRD500 m, summed length of paved roads in 500 m radius; PSUMRD1000 m, summed length of paved roads in 1000 m radius; DTWATER, distance to nearest surface water; DTSTRUC, distance to nearest human structure; (.), null model; PRECIP, weekly sum of precipitation during survey week; TEMP, weekly average temperature during survey week; ONROAD, whether or not a camera was placed on road (paved or unpaved).

b Akaike’s Information Criterion.

c Model weight.

d Number of model parameters.

e Difference in -2Log(Likelihood) of the current model and -2log(Likelihood) of the saturated model as a measure of model fit.

f Estimate of effect size.

g Standard error of effect size.

At urban camera sites, opossum was detected at the greatest proportion (89%) of sites, followed by raccoon at 78% of sites. Gray fox and striped skunk were both detected at 33% of urban sites. Coyote was detected at 22% and bobcat was detected at 17% of the urban cameras. At rural camera sites, raccoon was detected at the greatest proportion of sites, 79%. Bobcat and opossum were detected at 71% of sites while gray fox was detected at 50% of rural sites. Coyote and striped skunk were both detected at 29% of rural sites. In the MacDonald-Dunn Research Forest, striped skunk was detected at the most cameras, 53%. Bobcat and gray fox were detected at 46% and 40% of sites, respectively. Coyote and opossum were both detected at 33% of MacDonald-Dunn Research Forest, sites. Raccoon was detected at the lowest number of forested sites, 27%.

### Temporal activity patterns

**Figure 2:**
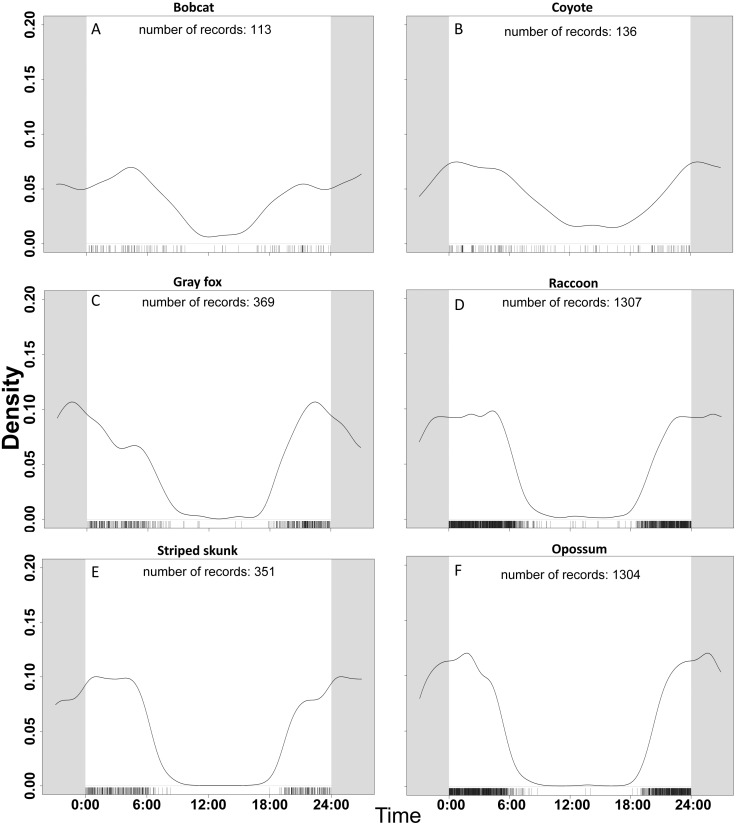
Temporal activity of six species based on data from 47 camera trap sites in and near Corvallis, OR, April 2018–February 2019. Comparisons of activity from 0:00 to 24:00 between (A) bobcat, (B) coyote, (C) gray fox, (D) raccoon, (E) striped skunk, and (F) opossum. Gray area continues the time cycle on either side of 0:00 and 24:00. The number of records reflects the number of detections per species once duplicate events (events within 30 min of each other) were removed. Mesocarnivores in the aggregate were more active during crepuscular and nighttime hours. Larger bodied mesocarnivores (bobcats, coyotes) were more active during the day than smaller bodied species (opossum, gray fox, raccoon, striped skunk).

For our temporal activity analysis, we used only detections that were greater than 30-minutes apart based on the findings of Ridout & Linkie (2009). Our focal species were detected most frequently during nocturnal hours, but the proportion of daylight detections varied by species (Fig. 2). Across all non-human species, 139 of 3580 detections (3.88%) were between 0800–1800. Out of 3589 detections of human, 3119 (86.9%) were between the hours of 0800-1800. Coyote and bobcat, the two largest of the six focal species, were the species most active during the day. These two species were also likely to favor natural areas over urbanized ones. The species that took advantage of urbanization, like raccoon and opossum, had much less daytime activity. Coyote had the most daytime activity relative to the other mesopredator species in our study with 25 out of 136 (18.4%) detections between 0800–1800. Striped skunk and opossum were the least likely to be detected during these hours with fewer than 2% of detections from 0800–1800. In general, the smaller species such as gray fox, opossum, raccoon, and striped skunk had much less diurnal activity overlap with larger species.

### Bobcat

Roads (*β*_ONROAD_ = 1.08 ± 0.25) were the strongest predictor of bobcat detection probability (Appendix A). Based on the most supported bobcat model [Ψ(UC100M+GC100M), *p* (ONROAD)], bobcat occupancy declined with urban cover (*β*_UC100M_ =  − 1.08 ± 0.43) and increased with grassland cover (*β*_GC100M_ = 0.79 ± 0.65) at the 100 m scale. The second most-supported model (ΔAIC_c_ = 0.23) was the univariate model with urban cover showing a negative association with landscape use (*β*_UC100M_ =  − 1.18 ± 0.41).

### Coyote

A negative association with precipitation (*β*_PRECIP_ =  − 0.54 ± 0.12) was our strongest detection model for coyote and was carried forward to the site-level occupancy models. Our strongest occupancy model for coyote [Ψ(USUMRD100M+WC500M), *p* (PRECIP)], associated increased landscape use with the amount of water cover (streams, lakes, wetlands) within 500 m (*β*_WC500M_ = 0.73 ± 0.39) and the density of unpaved roads within 100 m (*β*_USUM100M_ = 0.79 ± 0.37). The second-ranked (ΔAIC_c_ = 0.85) model [Ψ(WC500M+FC100M), *p* (PRECIP)] indicated increased use of areas with higher amounts of forest cover within 100 m (*β*_FC100M_ = 0.74 ± 0.37). We also found the positive use relationships with water, unpaved roads, and forest cover in our third strongest (ΔAIC_c_ = 1.75) model.

### Gray fox

Gray fox detection probability was best predicted by roads (*β*_ONROAD_ =  − 1.57 ± 0.27). Gray fox landscape use appeared to be most influenced by grassland cover based on our top model [Ψ(GC1000M), *p* (ONROAD)], which indicated a positive relationship with grassland cover within 1000 m (*β*_GC1000M_ = 1.19 ± 0.42). Our top models also suggested that gray fox landscape use was influenced by paved road density and urban cover (Table 2). The second strongest (ΔAIC_c_ = 0.87) model [Ψ(GC1000M+PSUMRD500M), p(ONROAD)] indicated weak support for a negative effect of paved road density (*β*_PSUMRD500M_ =  − 0.47 ± 0.38). Our model that included urban cover (ΔAIC_c_ = 2.50) [Ψ(GC1000M+UC500M), *p* (ONROAD)] showed that there was no clear effect on landscape use by urban cover (*β*_UC500M_ =  − 0.034 ± 0.350).

### Opossum

Temperature (*β*_TEMP_ = 0.21 ± 0.07) was the strongest detection model for opossum, providing a more supported model than precipitation (*β*_PRECIP_ =  − 0.11 ± 0.06) and the placement of cameras on roads (*β*_ONROAD_ =  − 0.09 ± 0.23). The strongest occupancy model [Ψ(rDTSTRUC), *p* (TEMP)] suggests that opossum occupancy probability declines as distance to human structure increases (*β*_DTSTRUC_ =  − 1.78 ± 0.66) (Table 2). The model with the next most support (ΔAIC_c_ = 0.15) [Ψ(DTSTRUC+UC100M), *p* (TEMP)] showed that urban cover (*β*_UC100M_ = 0.80 ± 0.57) also had a positive relationship with opossum occupancy, as well as reaffirming the previous model’s influence of human structures (*β*_DTSTRUC_ =  − 1.20 ± 0.62). Models for unpaved roads (ΔAIC_c_ = 0.47) [Ψ(USUMRD500M), *p* (TEMP)] and forest cover (ΔAIC_c_ = 0.51) [Ψ(FC100M), *p* (TEMP)] also showed to influence opossum landscape use. Both unpaved roads (*β*_USUMRD500M_ =  − 1.32 ± 0.41) and forest cover (*β*_FC100M_ =  − 1.32 ± 0.40) had a negative relationship to opossum occupancy.

### Raccoon

Our top racoon detection models suggested that roads negatively impacted detection (*β*_ONROAD_ =  − 2.80 ± 0.40) and had a greater magnitude of effect than precipitation (*β*_PRECIP_ = 0.06 ± 0.06) or temperature (*β*_TEMP_ =  − 0.1 ± 0.06). The strongest raccoon occupancy model was a univariate model [Ψ(rDTSTRUC), *p* (ONROAD)] with landscape use decreasing as distance to human structures increased (*β*_DTSTRUC_ =  − 1.57 ± 0.63) (Table 2). The second most supported (ΔAIC_c_ = 2.05) [Ψ(DTSTRUC+PSUMRD100M), *p* (ONROAD)] model indicated a weak positive relationship with paved road density (*β*_PSUMRD100M_ = 0.31 ± 0.47) along with a strong positive relationship between raccoon occupancy and human structures (*β*_DTSTRUC_ =  − 1.32 ± 0.69) (Table 2).

### Striped skunk

The most supported detection model for striped skunk included a negative effect of camera placement on roads (*β*_ONROAD_ =  − 0.67 ± 0.22), which we carried forward and used with all subsequent landscape occupancy models. The most supported use model [Ψ(FC500M), p(ONROAD)] for striped skunk included the positive effect of forest cover at 500 m (*β*_FC500M_ = 0.8753 ± 0.3370) (Table 2). The second strongest model (ΔAIC_c_ = 1.84), [Ψ(PSUMRD100M), *p* (ONROAD)], indicated a negative effect of paved road density at 100 m (*β*_SUMRD1000M_ =  − 0.8250 ± 0.3804). The third strongest model (ΔAIC_c_ = 2.06) [Ψ(UC1000M), *p* (ONROAD)] included negative relationship to urban cover at the 1000 m scale, (*β*_UC1000M_ =  − 0.82 ± 0.39).

## Discussion

We explored mesopredator occupancy in human-dominated landscapes across a gradient of urban, rural, and forested environments. Mammalian carnivores can be sensitive to urbanization and have varying responses along an urban to rural gradient (Goad et al., 2014; Salek, Drahnikova & Tkadlec, 2015; Wang, Allen & Wilmers, 2015). The species-level responses to anthropogenic disturbance varied tremendously, with some species being human exploiters tied to urban cover, others being human adapters that were neither positively nor negatively affected by disturbance and agriculture, and human avoider species that were negatively associated with anthropogenic landscape features. The individual species results of our study were largely supported by some established literature from other regions. However, some of our results were contradictory to expectations based on previous research. For example, we found that grassland cover was a more important landscape feature than forest cover for both bobcat and gray fox, whereas in other studies, forest cover was a better predictor of landscape use (Tucker, Clark & Gosselink, 2008; Lesmeister et al., 2015). We found that raccoon and opossum were human exploiters, and in contrast to other studies, striped skunk landscape use was negatively associated with urban development.

**Figure 3:**
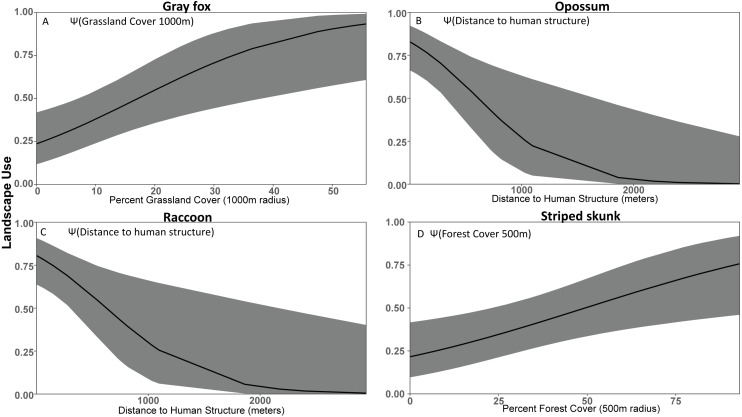
Covariate effect size plots for species with univariate models based on data from 47 camera trap sites in and near Corvallis, OR, April 2018–February 2019. Landscape use of (A) gray fox, (B) opossum, (C) raccoon, and (D) striped skunk using the most supported occupancy model for each respective species. The black line represents the use estimate while the shaded area represents the upper and lower confidence intervals.

We were able to identify opossum and raccoon as human exploiters due to their high use of urban cover and areas near structures (Fig. 3, Table 2). Opossum was common in both urban and rural developments, had negative relationships with forested cover, and increased use in areas with high urban cover density, suggesting that they can exploit much more densely populated areas than other species. Similarly, raccoon occupancy was greatest in urban areas and lowest in forested sites. This is consistent with previous results suggesting that opossum (Markovchick-Nicholls et al., 2008; Wright, Burt & Jackson, 2012) and raccoon (Prange, Gehrt & Wiggers, 2003; Prange, Gehrt & Wiggers, 2004) are competent cohabiters with humans that exploit urban areas. Although we do not have direct data on their population density, the patterns of occupancy that we observed strongly suggest that raccoon and opossum population density is substantially higher in urban areas. The occupancy of opossum and raccoon was strongly associated with human structures, and opossum was positively associated with urban cover even when accounting for proximity to human structures (Table 2). Similarly, opossum had a substantially stronger negative relationship with forest cover, suggesting that raccoon, unlike opossum, were not actively avoiding forested areas (Table 2). Collectively, these results suggest that while both species are associated with humans, opossum have greater affinity for urban environments. Presumably that affinity for urban environments is due to a combination of anthropogenic subsidies (Bozek, Prange & Gehrt, 2007) and reduced predation risk from larger predators (Lesmeister et al., 2015). The association of raccoon and opossum with urban environments was expected, but we did not find the predicted association with natural water sources that was found by others to be an important predictor of opossum and raccoon occupancy (Stuewer, 1943; Gehrt & Fritzell, 1998; Gehrt & Fritzell, 1999; Fidino, Lehrer & Magle, 2016). However, Fidino, Lehrer & Magle (2016) also noted that anthropogenic water sources may allow opossum to colonize previously uninhabitable urban patches. We did not include small human-derived water sources as a covariate in our models because they can be ephemeral and not detectable in land cover databases.

We expected striped skunk to be a human adapted species; but our results suggest that they are human avoiders with higher use of forest cover and low use of human development. Our findings are similar to some studies (Stout & Sonenshine, 1974; Bixler & Gittleman, 2000), but differ from other studies that found striped skunk home ranges oriented along roads and levees and that this species associates with structures for resting and denning (Frey & Conover, 2006; Theimer et al., 2017). Our study area experiences less volatile temperatures compared to those in the study areas of Frey & Conover (2006) and Theimer et al. (2017), which may mean less incentive to use human structures to help in thermoregulation. Dissuading depredation from larger species by their defense mechanism and warning coloration (Walton & Larivière, 1994; Fisher & Stankowich, 2018) may also make forested cover more viable for striped skunk compared to species of similar body size.

**Figure 4:**
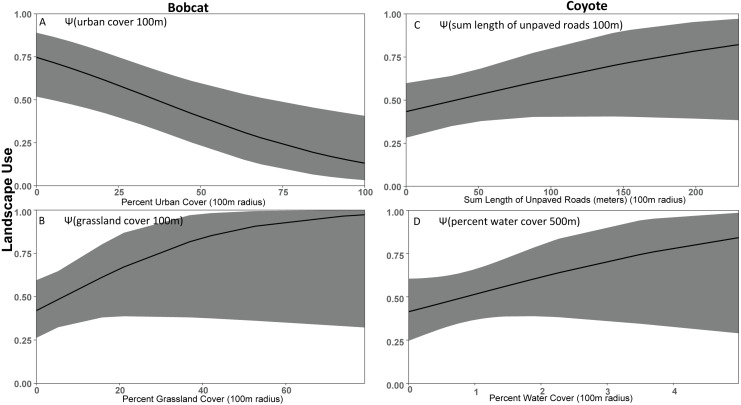
Covariate effect size plots for species with multivariate models based on data from 47 camera trap sites in and near Corvallis, OR, April 2018–February 2019. Bobcat landscape use based on urban cover at the 100 m scale (A) and grassland cover at the 100 m scale (B) and coyote landscape use on sum of unpaved roads at the 100 m scale (C) and water cover at the 100 m scale (D). The black line represents the use estimate while the shaded area represents the upper and lower confidence intervals.

As expected based on previous studies (Lesmeister et al., 2015), bobcat tended to be human avoiders in this study with a negative association with urban cover (Fig. 4). Previous studies have shown that when bobcat inhabit urbanized areas their home ranges consist primarily of natural areas (Riley et al., 2003; Riley, 2006). Bobcat appear to decline in response to increasing urbanization proximity and intensity (Tigas, Van Vuren & Sauvajot, 2002; George & Crooks, 2006; Ordeñana et al., 2010). Crooks (2002) suggests that bobcat appear to be particularly sensitive to fragmentation and the probability of bobcat occurrence is greater in larger reserves. In George & Crooks (2006) bobcat was less likely to be detected on cameras with high human activity. Cameras we placed in the Macdonald-Dunn Research Forest were on trails or roads that were commonly frequented by humans. If bobcat was avoiding areas with human activity, we may have observed a greater association with forest cover if we surveyed more forest sites away from human-use areas. We noted that while bobcat use was greater in less developed areas there were some detections in highly developed areas, although these events were infrequent similar to that of Goad et al. (2014).

Our results did not support our prediction that coyote would be human avoiders. Rather, our results suggest that coyote preferred natural areas like grassland and water cover, and urban features had little effect. Thusly, it was difficult to define coyote as solely human avoiders or exploiters, so we classified them as human adapters. We found that coyote were positively associated with unpaved roads, suggesting modest association with the lightest form of anthropogenic disturbance, but coyote was also positively associated with water and forest cover. Other studies have shown coyote frequently exploit urban environments due to their ability to take advantage of anthropogenic features for food, water, and shelter (Chamberlain, Lovell & Leopold, 2000; Kays, Gompper & Ray, 2008; Gehrt, Riley & Cypher, 2010). However, previous research also suggests that coyote need core wildland habitats (Grinder & Krausman, 2001; Gehrt, Anchor & White, 2009). There was modest support that forest cover was a positive predictor for coyote use, but that effect was less important than we predicted when compared to other covariates such as water cover (Riley et al., 2003; Tucker, Clark & Gosselink, 2008; Gehrt, Anchor & White, 2009; Magle et al., 2014).

In our study area, gray fox appeared to be neither strictly human exploiters nor avoiders. Following the precedent set by our coyote results and classification, we classified gray fox as human adapters. In the literature, there are conflicting gray fox results across various landscapes about how the species is positioned on the exploiter-avoider continuum. Results from Riley (2006) suggest that gray fox reach higher densities in and around urbanized areas, whereas Markovchick-Nicholls et al. (2008) found that gray fox were negatively associated with roads, much like our results. Lesmeister et al. (2015) noted that landscape use of gray fox may be driven by both interference competition by bobcat and coyote and the availability of small mammal prey. Our results suggested a negative relationship with human developments, but the support is weak enough that we did not feel it would be appropriate to classify this species as a human avoider in this study area. In contrast to results from Lesmeister et al. (2015), we found no evidence that forest cover was a significant predictor for gray fox occurrence, in either direction, but in our study area, free-ranging dogs were not as prevalent as in southern Illinois, which may have a depressing effect on gray fox landscape use (Morin et al., 2018). We observed that grassland cover was positively associated with gray fox occurrence, and previous studies found increased abundance of gray fox in areas with high levels of fragmentation of forested and grassland cover (Fuller & Cypher, 2004; Cooper, Nielsen & McDonald, 2012). Corvallis and the surrounding landscape have moderate levels of fragmentation from urban development, timber harvesting, and agricultural fields.

We have characterized the habitat associations of mesocarnivores, but mesocarnivore occupancy can also be influenced by competition and predation. We would be remiss to overlook the importance of cougars as the apex predator in carnivore communities. Throughout our survey effort, there were only 30 detections of cougar, and they were largely confined to the MacDonald-Dunn Research Forest. Based on previous studies (Chamberlain, Lovell & Leopold, 2000; O’connell et al., 2006; Cove et al., 2012; Lesmeister et al., 2015; Gompper et al., 2016), we expected forest cover to be a stronger predictor of landscape occupancy for mesopredators, but many of these studies occurred in areas without cougar where forested areas posed less risk. Species interactions with coyote may also be influencing the distribution of gray fox, which appear to be avoiding forested areas where coyote were more common to reduce intraguild conflict and interference competition (Temple, Chamberlain & Conner, 2010; Levi & Wilmers, 2012; Lesmeister et al., 2015). In another urban-rural carnivore study, raccoon distribution was negatively affected by coyote distribution while being positively correlated with predictors such as road density and distance to urban center (Randa & Yunger, 2006).

Our findings supported the predictions that mesopredators would have limited activity during daylight hours. Previous studies have also documented increased nocturnal activity by mesocarnivores (Riley et al., 2003; Wang, Allen & Wilmers, 2015). There appears to be a distinction between the activity patterns of species that preferred natural areas versus urban areas. However, we were unable to determine if this was primarily driven by body size or human activity, or if both were contributing factors. Both bobcat and coyote appeared to keep their circadian patterns as detections of them were common around dawn and dusk. Species with the most urban use, raccoon and opossum, showed less daytime activity (0600-1800) presumably to avoid increased human activity in these areas during these hours. Many studies (Larivière & Messier, 1998; Neiswenter, Dowler & Young, 2010) have documented the nocturnal nature of striped skunk, and that was reflected in our results as well.

## Conclusions

As urbanization continues, it is important to understand how a species might respond to increased urban development and the factors that might put a species at increased risk of decline to avoid the decline of once-common carnivores that has been observed in other species (Purvis et al., 2000; Gompper & Hackett, 2005; Gaston & Fuller, 2007). The taxa in this study have large ranges spanning much of North America, yet it is common for studies in different regions to observe a variety of species-specific relationships with land cover and human development. We provide information on how some common mesocarnivores use landscapes across a gradient of urban to rural to forested environments in the context of the vertebrate community and forest type of the mesic Pacific Northwest forest of the United States. Our results differed from several studies of this guild in other regions of North America, highlighting the importance of ecological context for landscape use along a gradient of urban to natural land cover. The level of human tolerance and species interactions may have also influenced our observed landscape use. For example, gray fox was typically associated with forest and grassland cover, but competitors such as bobcat (Lesmeister et al., 2015) and interference competition in areas of high coyote abundance can influence gray fox distribution (Crooks, 2002; Farias et al., 2005; Temple, Chamberlain & Conner, 2010; Ordeñana et al., 2010). Our results inform how mesopredator communities respond to anthropogenic disturbance in a distinct context, which together with other research can help elucidate the mechanisms underlying their observed distributions and inform their conservation and management. Management actions that impact these species are likely to be most effective if based on locally derived information.

##  Supplemental Information

10.7717/peerj.11083/supp-1 Supplemental Information 1 Site and survey level covarites across the study area in and near Corvallis, OR, April 2018 - February 2019 Covaraite values (forest, water, urban cover, etc.) of each site along with the temperature and precipitation data during the survey period.

10.7717/peerj.11083/supp-2 Supplemental Information 2 Binary detection histories for species in the Corvallis, OR, study area April 2018 - February 2019 Weekly binary detection histories for deer, coyote, cougar, domestic cat, domestic dog, gray fox, human, opossum, raccoon, and striped skunk.

10.7717/peerj.11083/supp-3 Supplemental Information 3 Species detection histories for activity analysis. Date and time data for deer, coyote, cougar, domestic cat, domestic dog, gray fox, human, opossum, raccoon, and striped skunk.

10.7717/peerj.11083/supp-4 Supplemental Information 4 Detection and occupancy code for for the species of interest with PRESENCE output files R version 3.53 (2019-03-11) used to run the provided code. ’RPresence’ version 2.12.27. ’ggplot2′version 3.3.1.

10.7717/peerj.11083/supp-5 Supplemental Information 5 Evaluation of survey covariates related to detection probabilities (*p*) for bobcats, coyotes, gray foxes, opossums, raccoons, and striped skunks in and near Corvallis, OR, April 2018 –February 2019 To estimate *p* for each species, we held the occupancy parameter constant and fit encounter history data from 47 camera sites. We included the null (.) model for each species for assessment of relative strength of survey covariates to explain heterogeneity in detection probabilities.

10.7717/peerj.11083/supp-6 Supplemental Information 6 Occupancy ( Ψ) model results from Single-Species Single-Season Models for bobcats, coyotes, gray foxes, opossums, raccoons, and striped skunks in and near Corvallis, OR, April 2018 –February 2019 To estimate Ψ for each species, we used the detection parameter that was most supported for the respective species and fit encounter history data from 47 camera sites.

## Additional Information and Declarations

### Field Study Permissions

The following information was supplied relating to field study approvals (i.e., approving body and any reference numbers): Access to field sites on private human dwellings were granted via verbal permission from private landowners; Alan Herlihy, Bill Gerth, Bill Pearcy, Bill Ripple, Brooke Penaluna & Ivan Arismendi, Chance Plunk, Cheryl Good, Daniel K. Rosenberg, David Culp, David Noakes, Emily Loose, Eric Forman, Frank Isaacs & Jane Olson, Howard Horton, Jana Seeliger, Jefferson Deweber, Jenna Baker, Jonny Armstrong, Judy Li, Julie Jenkins, Linda Ashkenas, Linsey Arnold & Peter Kappes, Marcia Snyder, Matt Konkler, Nancy Allen, Nathan Schumaker, Selina Heppell, Stephanie Clark, Susan Haig, Susie Dunham. Access to field sites on the MacDonald-Dunn Research Forest were granted via verbal permission from Oregon State University Research Forest managers Stephen Fitzgerald and Brent Klumpf.

### Data Availability

The following information was supplied regarding data availability: Raw data and code are available in the Supplemental Files.

## References

[ref-1] Akaike H (1974). A new look at the statistical model identification. IEEE Transactions on Automatic Control.

[ref-2] Aronson MFJ, La Sorte FA, Nilon CH, Katti M, Goddard MA, Lepczyk CA, Warren PS, Williams NSG, Cilliers S, Clarkson B, Dobbs C, Dolan R, Hedblom M, Klotz S, Kooijmans JL, Kühn I, MacGregor-Fors I, McDonnell M, Mörtberg U, Pyšek P, Siebert S, Sushinsky J, Werner P, Winter M (2014). A global analysis of the impacts of urbanization on bird and plant diversity reveals key anthropogenic drivers. Proceedings of the Royal Society B: Biological Sciences.

[ref-3] Atwood TC, Weeks HP, Gehring TM (2004). Spatial ecology of coyotes along a suburban-to-rural gradient. Journal of Wildlife Management.

[ref-4] Baker PJ, Harris S (2007). Urban mammals: what does the future hold? An analysis of the factors affecting patterns of use of residential gardens in Great Britain. Mammal Review.

[ref-5] Baker PJ, Harris S, Robertson CPJ, Saunders G, White PCL (2004). Is it possible to monitor mammal population changes from counts of road traffic casualties? An analysis using Bristol’s red foxes Vulpes vulpes as an example. Mammal Review.

[ref-6] Bateman PW, Fleming PA (2012). Big city life: carnivores in urban environments: Urban carnivores. Journal of Zoology.

[ref-7] Bixler A, Gittleman JL (2000). Variation in home range and use of habitat in the striped skunk (Mephitis mephitis). Journal of Zoology.

[ref-8] Blair RB (1996). Land use and avian species diversity along an urban gradient. Ecological Applications.

[ref-9] Blanchoud H, Farrugia F, Mouchel JM (2004). Pesticide uses and transfers in urbanised catchments. Chemosphere.

[ref-10] Bozek CK, Prange S, Gehrt SD (2007). The influence of anthropogenic resources on multi-scale habitat selection by raccoons. Urban Ecosystems.

[ref-11] Butchart SHM, Walpole M, Collen B, van Strien A, Scharlemann JPW, Almond REA, Baillie JEM, Bomhard B, Brown C, Bruno J, Carpenter KE, Carr GM, Chanson J, Chenery AM, Csirke J, Davidson NC, Dentener F, Foster M, Galli A, Galloway JN, Genovesi P, Gregory RD, Hockings M, Kapos V, Lamarque J-F, Leverington F, Loh J, McGeoch MA, McRae L, Minasyan A, Morcillo MH, Oldfield TEE, Pauly D, Quader S, Revenga C, Sauer JR, Skolnik B, Spear D, Stanwell-Smith D, Stuart SN, Symes A, Tierney M, Tyrrell TD, Vié J-C, Watson R (2010). Global Biodiversity: indicators of recent declines. Science.

[ref-12] Chamberlain MJ, Lovell CD, Leopold BD (2000). Spatial-use patterns, movements, and interactions among adult coyotes in central Mississippi. Canadian Journal of Zoology.

[ref-13] Clare JDJ, Anderson EM, Macfarland DM (2015). Predicting bobcat abundance at a landscape scale and evaluating occupancy as a density index in Central Wisconsin. Journal of Wildlife Management.

[ref-14] Cooper SE, Nielsen CK, McDonald PT (2012). Landscape factors affecting relative abundance of gray foxes Urocyon cinereoargenteus at large scales in Illinois, USA. Wildlife Biology.

[ref-15] Cove MV, Jones BM, Bossert AJ, Clever DR, Dunwoody RK, White BC, Jackson VL (2012). Use of camera traps to examine the mesopredator release hypothesis in a fragmented midwestern landscape. The American Midland Naturalist.

[ref-16] Crooks KR (2002). Relative sensitivities of mammalian carnivores to habitat fragmentation. Conservation Biology.

[ref-17] Crooks KR, Soulé ME (1999). Mesopredator release and avifaunal extinctions in a fragmented system. Nature.

[ref-18] Edson C, Wing GM (2011). Airborne light detection and ranging (LiDAR) for individual tree stem location, height, and biomass measurements. Remote Sensing.

[ref-19] Farias V, Fuller TK, Wayne RK, Sauvajot RM (2005). Survival and cause-specific mortality of gray foxes (Urocyon cinereoargenteus) in southern California. Journal of Zoology.

[ref-20] Fidino MA, Lehrer EW, Magle SB (2016). Habitat dynamics of the virginia opossum in a highly urban landscape. The American Midland Naturalist.

[ref-21] Fisher KA, Stankowich T (2018). Antipredator strategies of striped skunks in response to cues of aerial and terrestrial predators. Animal Behaviour.

[ref-22] Fletcher R, Johnson B, Blanchard G, Emmingham B, Hayes J, Johnson D, Johnson N, Lysne D, Murphy G, Newton M, Sessions J (2005). MacDonald-Dunn Forest Plan. Interdisciplinary Planning Team. https://cf.forestry.oregonstate.edu/sites/default/files/mcdunn_plan.pdf.

[ref-23] Flores-Morales M, Vázquez J, Bautista A, Rodríguez-Martínez L, Monroy-Vilchis O (2019). Response of two sympatric carnivores to human disturbances of their habitat: the bobcat and coyote. Mammal Research.

[ref-24] Frey SN, Conover MR (2006). Habitat use by meso-predators in a corridor environment. Journal of Wildlife Management.

[ref-25] Fuller TK, Cypher BL (2004). Gray fox: Urocyon cinereoargenteus. Canids: Foxes, wolves, jackals and dogs.

[ref-26] Gaston KJ, Fuller RA (2007). Biodiversity and extinction: losing the common and the widespread. Progress in Physical Geography: Earth and Environment.

[ref-27] Gaston K, Smith R, Thompson K, Warren P (2005). Urban domestic gardens (II): experimental tests of methods for increasing biodiversity. Biodiversity and Conservation.

[ref-28] Gehrt SD, Anchor C, White LA (2009). Home range and landscape use of coyotes in a metropolitan landscape: conflict or coexistence?. Journal of Mammalogy.

[ref-29] Gehrt SD, Fritzell EK (1998). Resource distribution, female home range dispersion and male spatial interactions: group structure in a solitary carnivore. Animal Behaviour.

[ref-30] Gehrt SD, Fritzell EK (1999). Behavioural aspects of the raccoon mating system: determinants of consortship success. Animal Behaviour.

[ref-31] Gehrt SD, Riley SPD, Cypher BL (2010). Urban carnivores: ecology, conflict, and conservation.

[ref-32] George SL, Crooks KR (2006). Recreation and large mammal activity in an urban nature reserve. Biological Conservation.

[ref-33] Goad EH, Pejchar L, Reed SE, Knight RL (2014). Habitat use by mammals varies along an exurban development gradient in northern Colorado. Biological Conservation.

[ref-34] Gompper ME, Hackett HM (2005). The long-term, range-wide decline of a once common carnivore: the eastern spotted skunk (Spilogale putorius). Animal Conservation.

[ref-35] Gompper ME, Lesmeister DB, Ray JC, Malcolm JR, Kays R (2016). Differential habitat use or intraguild interactions: What structures a carnivore community?. PLOS ONE.

[ref-36] Grinder MI, Krausman PR (2001). Home range, habitat use, and nocturnal activity of coyotes in an urban environment. The Journal of Wildlife Management.

[ref-37] Henner CM, Chamberlain MJ, Leopold BD, Burger LW (2004). A multi-resolution assessment of raccoon den selection. Journal of Wildlife Management.

[ref-38] Hines JE (2006). PRESENCE2- Software to estimate patch occupancy and related parameters. United States Geological Survey, Patuxent Wildlife Research Center. https://www.mbr-pwrc.usgs.gov/software/presence.html.

[ref-39] Hollings T, Jones M, Mooney N, McCallum H (2016). Disease-induced decline of an apex predator drives invasive dominated states and threatens biodiversity. Ecology.

[ref-40] Imhoff ML, Bounoua L, DeFries R, Lawrence WT, Stutzer D, Tucker CJ, Ricketts T (2004). The consequences of urban land transformation on net primary productivity in the United States. Remote Sensing of Environment.

[ref-41] Jetz W, Carbone C, Fulford J, Brown JH (2004). The scaling of animal space use. Science.

[ref-42] Kansky R, Knight AT (2014). Key factors driving attitudes towards large mammals in conflict with humans. Biological Conservation.

[ref-43] Kays RW, Gompper ME, Ray JC (2008). Landscape ecology of eastern coyotes based on large-scale estimates of abundance. Ecological Applications.

[ref-44] Kottek M, Grieser J, Beck C, Rudolf B, Rubel F (2006). World Map of the Köppen-Geiger climate classification updated. Meteorologische Zeitschrift.

[ref-45] Larivière S, Messier F (1998). Spatial organization of a prairie striped skunk population during the waterfowl nesting season. The Journal of Wildlife Management.

[ref-46] Lesmeister DB, Nielsen CK, Schauber EM, Hellgren EC (2015). Spatial and temporal structure of a mesocarnivore guild in midwestern north America: Midwestern Carnivore Guild Structure. Wildlife Monographs.

[ref-47] Levi T, Wilmers CC (2012). Wolves–coyotes–foxes: a cascade among carnivores. Ecology.

[ref-48] Lindstedt SL, Miller BJ, Buskirk SW (1986). Home range, time, and body size in mammals. Ecology.

[ref-49] Lowry H, Lill A, Wong BBM (2013). Behavioural responses of wildlife to urban environments. Biological Reviews.

[ref-50] Lucherini M, Reppucci J, Walker S, Villalba L, Wurstten A, Gallardo G, Iriarte A, Villalobos R, Perovic P (2009). Activity pattern segregation of carnivores in the high andes. Journal of Mammalogy.

[ref-51] Mackenzie DI, Nichols JD, Lachman GB, Droege S, Royle JA, Langtimm CA (2002). Estimating site occupancy rates when detection probabilities are less than one. Ecology.

[ref-52] MacKenzie DI, Nichols JD, Royle JA (2018). Occupancy estimation and modeling inferring patterns and dynamics of species occurrence.

[ref-53] Magle SB, Simoni LS, Lehrer EW, Brown JS (2014). Urban predator–prey association: coyote and deer distributions in the Chicago metropolitan area. Urban Ecosystems.

[ref-54] Markovchick-Nicholls L, Regan HM, Deutschman DH, Widyanata A, Martin B, Noreke L, Hunt TA (2008). Relationships between human disturbance and wildlife land use in urban habitat fragments. Conservation Biology.

[ref-55] Morin DJ, Lesmeister DB, Nielsen CK, Schauber EM (2018). The truth about cats and dogs: landscape composition and human occupation mediate the distribution and potential impact of non-native carnivores. Global Ecology and Conservation.

[ref-56] Morin DJ, Yackulic CB, Diffendorfer JE, Lesmeister DB, Nielsen CK, Reid J, Schauber EM (2020). Is your ad hoc model selection strategy affecting your multimodel inference?. *Ecosphere*.

[ref-57] Moss WE, Alldredge MW, Pauli JN (2016). Quantifying risk and resource use for a large carnivore in an expanding urban–wildland interface. Journal of Applied Ecology.

[ref-58] Neiswenter SA, Dowler RC, Young JH (2010). Activity patterns of two sympatric species of skunks (mephitis mephitis and spilogale gracilis) in texas. The Southwestern Naturalist.

[ref-59] Niedballa J, Courtiol A, Sollmann R, Mathai J, Wong ST, Nguyen ATT, Mohamed bin A, Tilker A, Wilting A (2016). camtrapR: an R package for efficient camera trap data management. Methods in Ecology and Evolution.

[ref-60] O’connell AF, Talancy NW, Bailey LL, Sauer JR, Cook R, Gilbert AT (2006). Estimating site occupancy and detection probability parameters for meso- and large mammals in a coastal ecosystem. Journal of Wildlife Management.

[ref-61] Ordeñana MA, Crooks KR, Boydston EE, Fisher RN, Lyren LM, Siudyla S, Haas CD, Harris S, Hathaway SA, Turschak GM, Miles AK, Van Vuren DH (2010). Effects of urbanization on carnivore species distribution and richness. Journal of Mammalogy.

[ref-62] Pickett STA, Cadenasso ML, Grove MJ, Nilon CH, Pouyat RV, Zipperer WC (2001). Urban ecological systems: linking terrestrial ecological, physical, and socioeconomic components of metropolitan areas. Annual Review of Ecological Systems.

[ref-63] Pimm SL, Jenkins CN, Abell R, Brooks TM, Gittleman JL, Joppa LN, Raven PH, Roberts CM, Sexton JO (2014). The biodiversity of species and their rates of extinction, distribution, and protection. Science.

[ref-64] Prange S, Gehrt SD, Wiggers EP (2003). Demographic factors contributing to high raccoon densities in urban landscapes. The Journal of Wildlife Management.

[ref-65] Prange S, Gehrt SD, Wiggers EP (2004). Influences of anthropogenic resources on raccoon (procyon lotor) movements and spatial distribution. Journal of Mammalogy.

[ref-66] Prugh LR, Stoner CJ, Epps CW, Bean WT, Ripple WJ, Laliberte AS, Brashares JS (2009). The rise of the mesopredator. BioScience.

[ref-67] Purvis A, Gittleman JL, Cowlishaw G, Mace GM (2000). Predicting extinction risk in declining species. Proceedings of the Royal Society of London. Series B: Biological Sciences.

[ref-68] Randa LA, Yunger JA (2006). Carnivore occurrence along an urban–rural gradient: a landscape-level analysis. Journal of Mammalogy.

[ref-69] Ridout M, Linkie M (2009). Estimating overlap of daily activity patterns from camera trap data. Journal of Agricultural Biological and Environmental Statistics.

[ref-70] Riggio J, Kija H, Masenga E, Mbwilo F, Van de Perre F, Caro T (2018). Sensitivity of Africa’s larger mammals to humans. Journal for Nature Conservation.

[ref-71] Riley SPD (2006). Spatial ecology of bobcats and gray foxes in urban and rural zones of a national park. Journal of Wildlife Management.

[ref-72] Riley SPD, Sauvajot RM, Fuller TK, York EC, Kamradt DA, Bromley C, Wayne RK (2003). Effects of urbanization and habitat fragmentation on bobcats and coyotes in Southern California. Conservation Biology.

[ref-73] Ritchie EG, Johnson CN (2009). Predator interactions, mesopredator release and biodiversity conservation. Ecology Letters.

[ref-74] Salek M, Drahnikova L, Tkadlec E (2015). Changes in home range sizes and population densities of carnivore species along the natural to urban habitat gradient. Mammal Review.

[ref-75] Santini L, González-Suárez M, Russo D, Gonzalez-Voyer Avon, Hardenberg A, Ancillotto L (2019). One strategy does not fit all: determinants of urban adaptation in mammals. Ecology Letters.

[ref-76] Schuette P, Wagner AP, Wagner ME, Creel S (2013). Occupancy patterns and niche partitioning within a diverse carnivore community exposed to anthropogenic pressures. Biological Conservation.

[ref-77] Seto KC, Guneralp B, Hutyra LR (2012). Global forecasts of urban expansion to 2030 and direct impacts on biodiversity and carbon pools. Proceedings of the National Academy of Sciences of the United States of America.

[ref-78] Soulsbury CD, White PCL (2016). Human–wildlife interactions in urban areas: a review of conflicts, benefits and opportunities. Wildlife Research.

[ref-79] Stout IJ, Sonenshine DE (1974). A striped skunk population in Virginia, 1963–69. Chesapeake Science.

[ref-80] Stuewer FW (1943). Raccoons: their habits and management in michigan. Ecological Monographs.

[ref-81] Temple D, Chamberlain M, Conner L (2010). Spatial ecology, survival and cause-specific mortality of gray foxes (*urocyon cinereoargenteus*) in a longleaf pine ecosystem. American Midland Naturalist - AMER MIDLAND NATURALIST.

[ref-82] Theimer TC, Williams CT, Johnson SR, Gilbert AT, Bergman DL, Buck CL (2017). Den use and heterothermy during winter in free-living, suburban striped skunks. Journal of Mammalogy.

[ref-83] Tigas LA, Van Vuren DH, Sauvajot RM (2002). Behavioral responses of bobcats and coyotes to habitat fragmentation and corridors in an urban environment. Biological Conservation.

[ref-84] Tucker SA, Clark WR, Gosselink TE (2008). Space use and habitat selection by bobcats in the fragmented landscape of South-Central Iowa. Journal of Wildlife Management.

[ref-85] US Census Bureau (2018). American FactFinder - Community Facts. https://factfinder.census.gov/faces/nav/jsf/pages/community_facts.xhtml.

[ref-86] US Climate Data US (1981-2010). Climate Corvallis - Oregon and Weather averages Corvallis. https://www.usclimatedata.com/climate/corvallis/oregon/united-states/usor0076/2019/1.

[ref-87] Walton LR, Larivière S (1994). A striped skunk, Mephitis mephitis, repels two coyotes, Canis latrans, without scenting. Canadian Field-Naturalist.

[ref-88] Wang Y, Allen ML, Wilmers CC (2015). Mesopredator spatial and temporal responses to large predators and human development in the Santa Cruz Mountains of California. Biological Conservation.

[ref-89] Woods M, Mcdonald RA, Harris S (2003). Predation of wildlife by domestic cats Felis catus in Great Britain. Mammal Review.

[ref-90] Wright JD, Burt MS, Jackson VL (2012). Influences of an urban environment on home range and body mass of virginia opossums (*Didelphis virginiana*). Northeastern Naturalist.

