# Supplementary material for: Mesocarnivore landscape use along a gradient of urban, rural, and forest cover
Source: PeerJ. 2021 Apr 6;9:e11083. doi: 10.7717/peerj.11083 (PMC8034353; doi:10.7717/peerj.11083)
Supplement: Supplemental Information 5 — To estimate p for each species, we held the occupancy parameter constant and fit encounter history data from 47 camera sites. We included the null (.) model for each species for assessment of relative strength of survey covariates to explain heterogeneity in detection probabilities. [file peerj-09-11083-s005.docx]

|  | Model^a^ | ΔAIC_c_ ^b^ | ω^c^ | K^d^ | neg2ll^e^ | β^f^ | se^g^ |
| --- | --- | --- | --- | --- | --- | --- | --- |
| Bobcat |  |  |  |  |  |  |  |
|  | ONROAD | 0.00 | 0.993 | 3 | 582.84 | 1.075 | 0.250 |
|  | TEMP | 10.16 | 0.006 | 3 | 593.00 | -0.341 | 0.120 |
|  | (.) | 16.22 | 0.000 | 2 | 601.34 | -0.051 | 0.303 |
|  | PRECIP | 16.91 | 0.000 | 3 | 599.75 | 0.130 | 0.101 |
| Coyote |  |  |  |  |  |  |  |
|  | PRECIP | 0.00 | 0.991 | 3 | 851.38 | -0.541 | 0.116 |
|  | TEMP | 9.50 | 0.009 | 3 | 860.89 | 0.393 | 0.092 |
|  | (.) | 26.26 | 0.000 | 2 | 879.92 | -1.229 | 0.087 |
|  | ONROAD | 27.17 | 0.000 | 3 | 878.56 | -0.223 | 0.193 |
| Gray Fox |  |  |  |  |  |  |  |
|  | ONROAD | 0.00 | 1.000 | 3 | 769.21 | -1.570 | 0.272 |
|  | (.) | 42.70 | 0.000 | 2 | 814.20 | -1.243 | 0.090 |
|  | TEMP | 43.88 | 0.000 | 3 | 813.09 | -0.096 | 0.091 |
|  | PRECIP | 44.73 | 0.000 | 3 | 813.94 | 0.043 | 0.084 |
| Opossum |  |  |  |  |  |  |  |
|  | TEMP | 0.00 | 0.946 | 3 | 1474.38 | 0.209 | 0.067 |
|  | PRECIP | 7.09 | 0.027 | 3 | 1481.47 | -0.108 | 0.064 |
|  | (.) | 7.73 | 0.020 | 2 | 1484.40 | -0.778 | 0.064 |
|  | ONROAD | 9.85 | 0.007 | 3 | 1484.24 | 0.092 | 0.227 |
| Raccoon |  |  |  |  |  |  |  |
|  | ONROAD | 0.00 | 1.000 | 3 | 1425.96 | -2.795 | 0.402 |
|  | TEMP | 97.44 | 0.000 | 3 | 1523.41 | -0.099 | 0.064 |
|  | (.) | 97.58 | 0.000 | 2 | 1525.83 | -0.555 | 0.062 |
|  | PRECIP | 98.97 | 0.000 | 3 | 1524.93 | 0.056 | 0.059 |
| Striped Skunk |  |  |  |  |  |  |  |
|  | ONROAD | 0.00 | 0.963 | 3 | 715.65 | -0.670 | 0.221 |
|  | (.) | 7.59 | 0.022 | 2 | 725.53 | -1.462 | 0.098 |
|  | TEMP | 9.45 | 0.009 | 3 | 725.10 | 0.064 | 0.098 |
|  | PRECIP | 9.81 | 0.007 | 3 | 725.46 | -0.023 | 0.093 |
| ^a^ PRECIP, weekly sum of precipitation during survey week; TEMP, weekly average temperature during survey week; ONROAD, whether or not a camera was placed on road (paved or unpaved). | | | | | | | |
| ^b^ Akaike’s Information Criterion. | | | | | |  |  |
| ^c^ Model weight. | | | | | |  |  |
| ^d^ Number of model parameters. | | | | | |  |  |
| ^e^ Difference in -2Log(Likelihood) of the current model and -2log(Likelihood) of the saturated model as a measure of model fit. | | | | | |  |  |
| ^f^ Estimate of effect size. | | | | | |  |  |
| ^g^ Standard error of effect size. | | | | | |  |  |
