# Supplementary material for: Mesocarnivore landscape use along a gradient of urban, rural, and forest cover
Source: PeerJ. 2021 Apr 6;9:e11083. doi: 10.7717/peerj.11083 (PMC8034353; doi:10.7717/peerj.11083)
Supplement: Supplemental Information 6 — To estimate Ψ for each species, we used the detection parameter that was most supported for the respective species and fit encounter history data from 47 camera sites. [file peerj-09-11083-s006.docx]

|  | Model^a^ | ΔAIC_c_^b^ | | ω^c^ | K^d^ | neg2ll^e^ |  | | β^f^ | | se^g^ |
| --- | --- | --- | --- | --- | --- | --- | --- | --- | --- | --- | --- |
| **Bobcat** |  | |  |  |  |  |  |  | |  | |
| FOREST COVER |  | |  |  |  |  |  |  | |  | |
|  | FC1000M | | 0.00 | 0.530 | 4 | 578.40 | Ψ(FC1000M) | 0.697 | | 0.348 | |
|  | (.) | | 2.04 | 0.190 | 3 | 582.84 | Ψ(.) | 0.047 | | 0.323 | |
|  | FC500M | | 2.10 | 0.180 | 4 | 580.50 | Ψ(FC500M) | 0.496 | | 0.333 | |
|  | FC100M | | 3.31 | 0.100 | 4 | 581.72 | Ψ(FC100M) | 0.342 | | 0.327 | |
| URBAN COVER |  | |  |  |  |  |  |  | |  | |
|  | UC100M | | 0.00 | 0.752 | 4 | 572.29 | Ψ(UC100M) | -1.184 | | 0.412 | |
|  | UC500M | | 2.72 | 0.193 | 4 | 575.01 | Ψ(UC500M) | -0.983 | | 0.381 | |
|  | UC1000M | | 5.76 | 0.042 | 4 | 578.05 | Ψ(UC1000M) | -0.780 | | 0.382 | |
|  | (.) | | 8.16 | 0.013 | 3 | 582.84 | Ψ(.) | 0.047 | | 0.323 | |
| GRASSLAND COVER | | |  |  |  |  |  |  | |  | |
|  | GC100M | | 0.00 | 0.419 | 4 | 577.50 | Ψ(GC100M) | 0.953 | | 0.561 | |
|  | GC500M | | 0.36 | 0.350 | 4 | 577.86 | Ψ(GC500M) | 0.872 | | 0.526 | |
|  | GC1000M | | 2.27 | 0.135 | 4 | 579.77 | Ψ(GC1000M) | 0.597 | | 0.376 | |
|  | (.) | | 2.94 | 0.096 | 3 | 582.84 | Ψ(.) | 0.047 | | 0.323 | |
| WATER COVER |  | |  |  |  |  |  |  | |  | |
|  | (.) | | 0.00 | 0.520 | 3 | 582.84 | Ψ(.) | 0.047 | | 0.323 | |
|  | WC500M | | 2.32 | 0.160 | 4 | 582.77 | Ψ(WC500M) | -0.086 | | 0.317 | |
|  | WC1000M | | 2.38 | 0.160 | 4 | 582.82 | Ψ(WC1000M) | -0.044 | | 0.319 | |
|  | WC100M | | 2.38 | 0.160 | 4 | 582.83 | Ψ(WC100M) | 0.035 | | 0.323 | |
| ROAD DENSITY UNPAVED | | |  |  |  |  |  |  | |  | |
|  | (.) | | 0.00 | 0.330 | 3 | 582.84 | Ψ(.) | 0.047 | | 0.323 | |
|  | USUMRD100M | | 0.45 | 0.270 | 4 | 580.89 | Ψ(USUMRD100M) | 0.443 | | 0.332 | |
|  | USUMRD500M | | 0.79 | 0.220 | 4 | 581.24 | Ψ(USUMRD500M) | 0.402 | | 0.328 | |
|  | USUMRD1000M | | 1.26 | 0.180 | 4 | 581.71 | Ψ(USUMRD1000M) | 0.351 | | 0.344 | |
| ROAD DENSITY PAVED | | |  |  |  |  |  |  | |  | |
|  | PSUMRD1000M | | 0.00 | 0.706 | 4 | 574.90 | Ψ(PSUMRD1000M) | -1.025 | | 0.402 | |
|  | PSUMRD500M | | 2.64 | 0.189 | 4 | 577.54 | Ψ(PSUMRD500M) | -0.816 | | 0.377 | |
|  | PSUMRD100M | | 4.90 | 0.061 | 4 | 579.80 | Ψ(PSUMRD100M) | -0.588 | | 0.351 | |
|  | (.) | | 5.54 | 0.044 | 3 | 582.84 | Ψ(.) | 0.047 | | 0.323 | |
| DISTANCE TO SURFACE WATER | | |  |  |  |  |  |  | |  | |
|  | (.) | | 0.00 | 0.650 | 3 | 582.84 | Ψ(.) | 0.047 | | 0.323 | |
|  | DTWATER | | 1.24 | 0.350 | 4 | 581.69 | Ψ(DTWATER) | -0.352 | | 0.333 | |
| DISTANCE TO HUMAN STRUCTURE | | |  |  |  |  |  |  | |  | |
|  | DTSTRUC | | 0.00 | 0.520 | 4 | 580.31 | Ψ(DTSTRUC) | 0.395 | | 0.260 | |
|  | (.) | | 0.14 | 0.480 | 3 | 582.84 | Ψ(.) | 0.047 | | 0.323 | |
| TOP MODELS (univariate) | | |  |  |  |  |  |  | |  | |
|  | UC100M | | 0.00 | 0.693 | 4 | 572.29 | Ψ(UC100M) | -1.184 | | 0.412 | |
|  | PSUMRD1000M | | 2.61 | 0.188 | 4 | 574.90 | Ψ(PSUMRD1000M) | -1.025 | | 0.402 | |
|  | GC100M | | 5.21 | 0.051 | 4 | 577.50 | Ψ(GC100M) | 0.953 | | 0.561 | |
|  | FC1000M | | 6.11 | 0.033 | 4 | 578.40 | Ψ(FC1000M) | 0.697 | | 0.348 | |
|  | (.) | | 8.16 | 0.012 | 3 | 582.84 | Ψ(.) | 0.047 | | 0.323 | |
|  | USUMRD100M | | 8.60 | 0.009 | 4 | 580.89 | Ψ(USUMRD100M) | 0.443 | | 0.332 | |
|  | DTWATER | | 9.40 | 0.006 | 4 | 581.69 | Ψ(DTWATER) | -0.352 | | 0.333 | |
|  | DTSTRUC | | 9.92 | 0.005 | 4 | 582.21 | Ψ(DTSTRUC) | 0.395 | | 0.260 | |
|  | WC500M | | 10.48 | 0.004 | 4 | 582.77 | Ψ(WC500M) | -0.086 | | 0.317 | |
| TOP MODELS (multivariate) | | |  |  |  |  |  |  | |  | |
|  | UC100M+GC100M | | 0.00 | 0.604 | 5 | 569.55 | Ψ(UC100M) | -1.080 | | 0.430 | |
|  |  | |  |  |  |  | Ψ(GC100M) | 0.786 | | 0.645 | |
|  | GC100M+FC1000M | | 2.21 | 0.200 | 5 | 571.76 | Ψ(GC100M) | 1.139 | | 0.615 | |
|  |  | |  |  |  |  | Ψ(FC1000M) | 0.811 | | 0.359 | |
|  | PSUMRD1000M+GC100M | | 2.35 | 0.187 | 5 | 571.90 | Ψ(PSUMRD1000M) | -0.887 | | 0.406 | |
|  |  | |  |  |  |  | Ψ(GC100M) | 0.728 | | 0.536 | |
|  | (.) | | 8.38 | 0.009 | 3 | 582.84 | Ψ(.) | 0.047 | | 0.323 | |
| ALL TOP MODELS | | |  |  |  |  |  |  | |  | |
|  | UC100M+GC100M | | 0.00 | 0.342 | 5 | 569.55 | Ψ(UC100M) | -1.080 | | 0.430 | |
|  |  | |  |  |  |  | Ψ(GC100M) | 0.786 | | 0.645 | |
|  | UC100M | | 0.23 | 0.305 | 4 | 572.29 | Ψ(UC100M) | -1.184 | | 0.412 | |
|  | GC100M+FC1000M | | 2.21 | 0.113 | 5 | 571.76 | Ψ(GC100M) | 1.139 | | 0.615 | |
|  |  | |  |  |  |  | Ψ(FC1000M) | 0.811 | | 0.359 | |
|  | PSUMRD1000M+GC100M | | 2.35 | 0.106 | 5 | 571.90 | Ψ(PSUMRD1000M) | -0.887 | | 0.406 | |
|  |  | |  |  |  |  | Ψ(GC100M) | 0.728 | | 0.536 | |
|  | PSUMRD1000M | | 2.84 | 0.083 | 4 | 574.90 | Ψ(PSUMRD1000M) | -1.025 | | 0.402 | |
|  | GC100M | | 5.44 | 0.023 | 4 | 577.50 | Ψ(GC100M) | 0.953 | | 0.561 | |
|  | FC1000M | | 6.34 | 0.014 | 4 | 578.40 | Ψ(FC1000M) | 0.697 | | 0.348 | |
|  | (.) | | 8.38 | 0.005 | 3 | 582.84 | Ψ(.) | 0.047 | | 0.323 | |
|  | USUMRD100M | | 8.83 | 0.004 | 4 | 580.89 | Ψ(USUMRD100M) | 0.443 | | 0.332 | |
|  | DTWATER | | 9.63 | 0.003 | 4 | 581.69 | Ψ(DTWATER) | -0.352 | | 0.333 | |
|  | DTSTRUC | | 10.15 | 0.002 | 4 | 582.21 | Ψ(DTSTRUC) | 0.395 | | 0.260 | |
|  | WC500M | | 10.70 | 0.002 | 4 | 582.77 | Ψ(WC500M) | -0.086 | | 0.317 | |
| **Coyote** |  | |  |  |  |  |  |  | |  | |
| FOREST COVER |  | |  |  |  |  |  |  | |  | |
|  | (.) | | 0.00 | 0.360 | 3 | 851.38 | Ψ(.) | 0.005 | | 0.296 | |
|  | FC100M | | 0.56 | 0.270 | 4 | 849.55 | Ψ(FC100M) | 0.419 | | 0.315 | |
|  | FC1000M | | 1.25 | 0.190 | 4 | 850.24 | Ψ(FC1000M) | 0.330 | | 0.313 | |
|  | FC500M | | 1.35 | 0.180 | 4 | 850.34 | Ψ(FC500M) | 0.312 | | 0.309 | |
| URBAN COVER |  | |  |  |  |  |  |  | |  | |
|  | (.) | | 0.00 | 0.400 | 3 | 851.38 | Ψ(.) | 0.005 | | 0.296 | |
|  | UC100M | | 0.56 | 0.300 | 4 | 849.55 | Ψ(UC100M) | -0.414 | | 0.310 | |
|  | UC500M | | 1.88 | 0.160 | 4 | 850.87 | Ψ(UC500M) | -0.217 | | 0.304 | |
|  | UC1000M | | 2.07 | 0.140 | 4 | 851.06 | Ψ(UC1000M) | -0.171 | | 0.303 | |
| GRASSLAND COVER | | |  |  |  |  |  |  | |  | |
|  | (.) | | 0.00 | 0.480 | 3 | 851.38 | Ψ(.) | 0.005 | | 0.296 | |
|  | GC1000M | | 1.60 | 0.220 | 4 | 850.59 | Ψ(GC1000M) | 0.268 | | 0.305 | |
|  | GC500M | | 2.33 | 0.150 | 4 | 851.32 | Ψ(GC500M) | 0.076 | | 0.298 | |
|  | GC100M | | 2.38 | 0.150 | 4 | 851.37 | Ψ(GC100M) | -0.038 | | 0.296 | |
| WATER COVER |  | |  |  |  |  |  |  | |  | |
|  | (.) | | 0.00 | 0.330 | 3 | 851.38 | Ψ(.) | 0.005 | | 0.296 | |
|  | WC500M | | 0.42 | 0.270 | 4 | 849.41 | Ψ(WC500M) | 0.444 | | 0.336 | |
|  | WC1000M | | 0.90 | 0.210 | 4 | 849.88 | Ψ(WC1000M) | 0.393 | | 0.341 | |
|  | WC100M | | 1.21 | 0.180 | 4 | 850.20 | Ψ(WC100M) | 0.348 | | 0.340 | |
| ROAD DENSITY UNPAVED | | |  |  |  |  |  |  | |  | |
|  | USUMRD100M | | 0.00 | 0.405 | 4 | 848.44 | Ψ(USUMRD100M) | 0.545 | | 0.340 | |
|  | (.) | | 0.55 | 0.308 | 3 | 851.38 | Ψ(.) | 0.005 | | 0.296 | |
|  | USUMRD500M | | 1.54 | 0.188 | 4 | 849.98 | Ψ(USUMRD500M) | 0.359 | | 0.311 | |
|  | USUMRD1000M | | 2.81 | 0.099 | 4 | 851.26 | Ψ(USUMRD1000M) | 0.105 | | 0.298 | |
| ROAD DENSITY PAVED | | |  |  |  |  |  |  | |  | |
|  | (.) | | 0.00 | 0.340 | 3 | 851.38 | Ψ(.) | 0.005 | | 0.296 | |
|  | PSUMRD100M | | 0.71 | 0.240 | 4 | 849.69 | Ψ(PSUMRD100M) | -0.396 | | 0.312 | |
|  | PSUMRD500M | | 0.74 | 0.230 | 4 | 849.72 | Ψ(PSUMRD500M) | -0.397 | | 0.314 | |
|  | PSUMRD1000M | | 1.11 | 0.190 | 4 | 850.10 | Ψ(PSUMRD1000M) | -0.346 | | 0.309 | |
| DISTANCE TO SURFACE WATER | | |  |  |  |  |  |  | |  | |
|  | (.) | | 0.00 | 0.770 | 3 | 851.38 | Ψ(.) | 0.005 | | 0.296 | |
|  | DTWATER | | 2.39 | 0.230 | 4 | 851.38 | Ψ(DTWATER) | -0.005 | | 0.299 | |
| DISTANCE TO HUMAN STRUCTURE | | |  |  |  |  |  |  | |  | |
|  | (.) | | 0.00 | 0.760 | 3 | 851.38 | Ψ(.) | 0.005 | | 0.296 | |
|  | DTSTRUC | | 2.31 | 0.240 | 4 | 851.30 | Ψ(DTSTRUC) | 0.085 | | 0.299 | |
| TOP MODELS (univariate) | | |  |  |  |  |  |  | |  | |
|  | USUMRD100M | | 0.00 | 0.205 | 4 | 848.44 | Ψ(USUMRD100M) | 0.545 | | 0.340 | |
|  | (.) | | 0.55 | 0.156 | 3 | 851.38 | Ψ(.) | 0.005 | | 0.296 | |
|  | WC500M | | 0.96 | 0.127 | 4 | 849.41 | Ψ(WC500M) | 0.444 | | 0.336 | |
|  | UC100M | | 1.10 | 0.118 | 4 | 849.55 | Ψ(UC100M) | -0.414 | | 0.310 | |
|  | FC100M | | 1.11 | 0.118 | 4 | 849.55 | Ψ(FC100M) | 0.419 | | 0.315 | |
|  | PSUMRD100M | | 1.25 | 0.110 | 4 | 849.69 | Ψ(PSUMRD100M) | -0.396 | | 0.312 | |
|  | GC1000M | | 2.15 | 0.070 | 4 | 850.59 | Ψ(GC1000M) | 0.268 | | 0.305 | |
|  | DTSTRUC | | 2.86 | 0.049 | 4 | 851.30 | Ψ(DTSTRUC) | 0.085 | | 0.299 | |
|  | DTWATER | | 2.94 | 0.047 | 4 | 851.38 | Ψ(DTWATER) | -0.005 | | 0.299 | |
| TOP MODELS (multivariate) | | |  |  |  |  |  |  | |  | |
|  | USUMRD100M+WC500M | | 0.00 | 0.183 | 5 | 844.12 | Ψ(USUMRD100M) | 0.785 | | 0.374 | |
|  |  | |  |  |  |  | Ψ(WC500M) | 0.729 | | 0.394 | |
|  | WC500M+FC100M | | 0.85 | 0.119 | 5 | 844.97 | Ψ(WC500M) | 0.781 | | 0.416 | |
|  |  | |  |  |  |  | Ψ(FC100M) | 0.740 | | 0.370 | |
|  | USUMRD100M+WC500M+FC100M | | 1.75 | 0.076 | 6 | 843.23 | Ψ(USUMRD100M) | 0.554 | | 0.437 | |
|  |  | |  |  |  |  | Ψ(WC500M) | 0.832 | | 0.423 | |
|  |  | |  |  |  |  | Ψ(FC100M) | 0.407 | | 0.437 | |
|  | USUMRD100M+WC500M+UC100M | | 2.22 | 0.060 | 6 | 843.70 | Ψ(USUMRD100M) | 0.676 | | 0.403 | |
|  |  | |  |  |  |  | Ψ(WC500M) | 0.742 | | 0.405 | |
|  |  | |  |  |  |  | Ψ(UC100M) | -0.233 | | 0.364 | |
|  | USUMRD100M+WC500M+GC1000M | | 2.32 | 0.057 | 6 | 843.80 | Ψ(USUMRD100M) | 0.800 | | 0.377 | |
|  |  | |  |  |  |  | Ψ(WC500M) | 0.678 | | 0.407 | |
|  |  | |  |  |  |  | Ψ(GC1000M) | 0.190 | | 0.338 | |
|  | (.) | | 2.36 | 0.056 | 3 | 851.38 | Ψ(.) | 0.005 | | 0.296 | |
|  | USUMRD100M+WC500M+PSUMRD100M | | 2.51 | 0.052 | 6 | 843.99 | Ψ(USUMRD100M) | 0.716 | | 0.417 | |
|  |  | |  |  |  |  | Ψ(WC500M) | 0.724 | | 0.397 | |
|  |  | |  |  |  |  | Ψ(PSUMRD100M) | -0.131 | | 0.365 | |
|  | USUMRD100M+PSUMRD100M+WC500M | | 2.51 | 0.052 | 6 | 843.99 | Ψ(USUMRD100M) | 0.716 | | 0.417 | |
|  |  | |  |  |  |  | Ψ(PSUMRD100M) | -0.131 | | 0.365 | |
|  |  | |  |  |  |  | Ψ(WC500M) | 0.724 | | 0.397 | |
|  | WC500M+UC100M | | 2.84 | 0.044 | 5 | 846.96 | Ψ(WC500M) | 0.549 | | 0.374 | |
|  |  | |  |  |  |  | Ψ(UC100M) | -0.499 | | 0.327 | |
|  | USUMRD100M+GC1000M | | 2.97 | 0.042 | 5 | 847.09 | Ψ(USUMRD100M) | 0.605 | | 0.349 | |
|  |  | |  |  |  |  | Ψ(GC1000M) | 0.358 | | 0.311 | |
|  | WC500M+FC100M+GC1000M | | 3.19 | 0.037 | 6 | 844.67 | Ψ(WC500M) | 0.729 | | 0.430 | |
|  |  | |  |  |  |  | Ψ(FC100M) | 0.754 | | 0.373 | |
|  |  | |  |  |  |  | Ψ(GC1000M) | 0.187 | | 0.340 | |
|  | WC500M+PSUMRD100M | | 3.24 | 0.036 | 5 | 847.36 | Ψ(WC500M) | 0.507 | | 0.359 | |
|  |  | |  |  |  |  | Ψ(PSUMRD100M) | -0.449 | | 0.324 | |
|  | USUMRD100M+UC100M | | 3.87 | 0.026 | 5 | 847.99 | Ψ(USUMRD100M) | 0.439 | | 0.369 | |
|  |  | |  |  |  |  | Ψ(UC100M) | -0.230 | | 0.343 | |
|  | USUMRD100M+PSUMRD100M | | 4.03 | 0.024 | 5 | 848.15 | Ψ(USUMRD100M) | 0.451 | | 0.379 | |
|  |  | |  |  |  |  | Ψ(PSUMRD100M) | -0.188 | | 0.349 | |
|  | FC100M+GC100M | | 4.07 | 0.024 | 5 | 848.18 | Ψ(FC100M) | 0.494 | | 0.326 | |
|  |  | |  |  |  |  | Ψ(GC1000M) | 0.364 | | 0.318 | |
|  | USUMRD100M+FC100M | | 4.20 | 0.022 | 5 | 848.32 | Ψ(USUMRD100M) | 0.453 | | 0.423 | |
|  |  | |  |  |  |  | Ψ(FC100M) | 0.142 | | 0.400 | |
|  | WC500M+GC1000M | | 5.11 | 0.014 | 5 | 849.22 | Ψ(WC500M) | 0.398 | | 0.355 | |
|  |  | |  |  |  |  | Ψ(GC1000M) | 0.139 | | 0.328 | |
|  | UC100M+GC1000M | | 5.11 | 0.014 | 5 | 849.23 | Ψ(UC100M) | -0.369 | | 0.319 | |
|  |  | |  |  |  |  | Ψ(GC1000M) | 0.179 | | 0.318 | |
|  | PSUMRD100M+GC1000M | | 5.15 | 0.014 | 5 | 849.26 | Ψ(PSUMRD100M) | -0.360 | | 0.319 | |
|  |  | |  |  |  |  | Ψ(GC1000M) | 0.203 | | 0.312 | |
|  | WC500M+UC100M+GC1000M | | 5.47 | 0.012 | 6 | 846.95 | Ψ(WC500M) | 0.560 | | 0.401 | |
|  |  | |  |  |  |  | Ψ(UC100M) | -0.507 | | 0.345 | |
|  |  | |  |  |  |  | Ψ(GC1000M) | -0.026 | | 0.352 | |
|  | USUMRD100M+UC100M+GC1000M | | 5.53 | 0.012 | 6 | 847.01 | Ψ(USUMRD100M) | 0.553 | | 0.395 | |
|  |  | |  |  |  |  | Ψ(UC100M) | -0.099 | | 0.371 | |
|  |  | |  |  |  |  | Ψ(GC1000M) | 0.326 | | 0.333 | |
|  | USUMRD100M+PSUMRD100M+GC1000M | | 5.57 | 0.011 | 6 | 847.05 | Ψ(USUMRD100M) | 0.566 | | 0.401 | |
|  |  | |  |  |  |  | Ψ(PSUMRD100M) | -0.072 | | 0.370 | |
|  |  | |  |  |  |  | Ψ(GC1000M) | 0.339 | | 0.326 | |
|  | WC500M+PSUMRD100M+GC1000M | | 5.87 | 0.010 | 6 | 847.35 | Ψ(WC500M) | 0.494 | | 0.379 | |
|  |  | |  |  |  |  | Ψ(PSUMRD100M) | -0.442 | | 0.331 | |
|  |  | |  |  |  |  | Ψ(GC1000M) | 0.036 | | 0.341 | |
| ALL TOP MODELS | | |  |  |  |  |  |  | |  | |
|  | USUMRD100M+WC500M | | 0.00 | 0.140 | 5 | 844.12 | Ψ(USUMRD100M) | 0.785 | | 0.374 | |
|  |  | |  |  |  |  | Ψ(WC500M) | 0.729 | | 0.394 | |
|  | WC500M+FC100M | | 0.85 | 0.092 | 5 | 844.97 | Ψ(WC500M) | 0.781 | | 0.416 | |
|  |  | |  |  |  |  | Ψ(FC100M) | 0.740 | | 0.370 | |
|  | USUMRD100M+WC500M+FC100M | | 1.75 | 0.059 | 6 | 843.23 | Ψ(USUMRD100M) | 0.554 | | 0.437 | |
|  |  | |  |  |  |  | Ψ(WC500M) | 0.832 | | 0.423 | |
|  |  | |  |  |  |  | Ψ(FC100M) | 0.407 | | 0.437 | |
|  | USUMRD100M | | 1.82 | 0.057 | 4 | 848.44 | Ψ(USUMRD100M) | 0.545 | | 0.340 | |
|  | USUMRD100M+WC500M+UC100M | | 2.22 | 0.046 | 6 | 843.70 | Ψ(USUMRD100M) | 0.676 | | 0.403 | |
|  |  | |  |  |  |  | Ψ(WC500M) | 0.742 | | 0.405 | |
|  |  | |  |  |  |  | Ψ(UC100M) | -0.233 | | 0.364 | |
|  | USUMRD100M+WC500M+GC1000M | | 2.32 | 0.044 | 6 | 843.80 | Ψ(USUMRD100M) | 0.800 | | 0.377 | |
|  |  | |  |  |  |  | Ψ(WC500M) | 0.678 | | 0.407 | |
|  |  | |  |  |  |  | Ψ(GC1000M) | 0.190 | | 0.338 | |
|  | (.) | | 2.36 | 0.043 | 3 | 851.38 | Ψ(.) | 0.005 | | 0.296 | |
|  | USUMRD100M+WC500M+PSUMRD100M | | 2.51 | 0.040 | 6 | 843.99 | Ψ(USUMRD100M) | 0.716 | | 0.417 | |
|  |  | |  |  |  |  | Ψ(WC500M) | 0.724 | | 0.397 | |
|  |  | |  |  |  |  | Ψ(PSUMRD100M) | -0.131 | | 0.365 | |
|  | USUMRD100M+PSUMRD100M+WC500M | | 2.51 | 0.040 | 6 | 843.99 | Ψ(USUMRD100M) | 0.716 | | 0.417 | |
|  |  | |  |  |  |  | Ψ(PSUMRD100M) | -0.131 | | 0.365 | |
|  |  | |  |  |  |  | Ψ(WC500M) | 0.724 | | 0.397 | |
|  | WC500M | | 2.78 | 0.035 | 4 | 849.41 | Ψ(WC500M) | 0.444 | | 0.336 | |
|  | WC500M+UC100M | | 2.84 | 0.034 | 5 | 846.96 | Ψ(WC500M) | 0.549 | | 0.374 | |
|  |  | |  |  |  |  | Ψ(UC100M) | -0.499 | | 0.327 | |
|  | UC100M | | 2.92 | 0.033 | 4 | 849.55 | Ψ(UC100M) | -0.414 | | 0.310 | |
|  | FC100M | | 2.92 | 0.033 | 4 | 849.55 | Ψ(FC100M) | 0.419 | | 0.315 | |
|  | USUMRD100M+GC1000M | | 2.97 | 0.032 | 5 | 847.09 | Ψ(USUMRD100M) | 0.605 | | 0.349 | |
|  |  | |  |  |  |  | Ψ(GC1000M) | 0.358 | | 0.311 | |
|  | PSUMRD100M | | 3.07 | 0.030 | 4 | 849.69 | Ψ(PSUMRD100M) | -0.396 | | 0.312 | |
|  | WC500M+FC100M+GC1000M | | 3.19 | 0.029 | 6 | 844.67 | Ψ(WC500M) | 0.729 | | 0.430 | |
|  |  | |  |  |  |  | Ψ(FC100M) | 0.754 | | 0.373 | |
|  |  | |  |  |  |  | Ψ(GC1000M) | 0.187 | | 0.340 | |
|  | WC500M+PSUMRD100M | | 3.24 | 0.028 | 5 | 847.36 | Ψ(WC500M) | 0.507 | | 0.359 | |
|  |  | |  |  |  |  | Ψ(PSUMRD100M) | -0.449 | | 0.324 | |
|  | USUMRD100M+UC100M | | 3.87 | 0.020 | 5 | 847.99 | Ψ(USUMRD100M) | 0.439 | | 0.369 | |
|  |  | |  |  |  |  | Ψ(UC100M) | -0.230 | | 0.343 | |
|  | GC1000M | | 3.96 | 0.019 | 4 | 850.59 | Ψ(GC1000M) | 0.268 | | 0.305 | |
|  | USUMRD100M+PSUMRD100M | | 4.03 | 0.019 | 5 | 848.15 | Ψ(USUMRD100M) | 0.451 | | 0.379 | |
|  |  | |  |  |  |  | Ψ(PSUMRD100M) | -0.188 | | 0.349 | |
|  | FC100M+GC100M | | 4.07 | 0.018 | 5 | 848.18 | Ψ(FC100M) | 0.494 | | 0.326 | |
|  |  | |  |  |  |  | Ψ(GC1000M) | 0.364 | | 0.318 | |
|  | USUMRD100M+FC100M | | 4.20 | 0.017 | 5 | 848.32 | Ψ(USUMRD100M) | 0.453 | | 0.423 | |
|  |  | |  |  |  |  | Ψ(FC100M) | 0.142 | | 0.400 | |
|  | DTSTRUC | | 4.67 | 0.014 | 4 | 851.30 | Ψ(DTSTRUC) | 0.085 | | 0.299 | |
|  | DTWATER | | 4.76 | 0.013 | 4 | 851.38 | Ψ(DTWATER) | -0.005 | | 0.299 | |
|  | WC500M+GC1000M | | 5.11 | 0.011 | 5 | 849.22 | Ψ(WC500M) | 0.398 | | 0.355 | |
|  |  | |  |  |  |  | Ψ(GC1000M) | 0.139 | | 0.328 | |
|  | UC100M+GC1000M | | 5.11 | 0.011 | 5 | 849.23 | Ψ(UC100M) | -0.369 | | 0.319 | |
|  |  | |  |  |  |  | Ψ(GC1000M) | 0.179 | | 0.318 | |
|  | PSUMRD100M+GC1000M | | 5.15 | 0.011 | 5 | 849.26 | Ψ(PSUMRD100M) | -0.360 | | 0.319 | |
|  |  | |  |  |  |  | Ψ(GC1000M) | 0.203 | | 0.312 | |
|  | WC500M+UC100M+GC1000M | | 5.47 | 0.009 | 6 | 846.95 | Ψ(WC500M) | 0.560 | | 0.401 | |
|  |  | |  |  |  |  | Ψ(UC100M) | -0.507 | | 0.345 | |
|  |  | |  |  |  |  | Ψ(GC1000M) | -0.026 | | 0.352 | |
|  | USUMRD100M+UC100M+GC1000M | | 5.53 | 0.009 | 6 | 847.01 | Ψ(USUMRD100M) | 0.553 | | 0.395 | |
|  |  | |  |  |  |  | Ψ(UC100M) | -0.099 | | 0.371 | |
|  |  | |  |  |  |  | Ψ(GC1000M) | 0.326 | | 0.333 | |
|  | USUMRD100M+PSUMRD100M+GC1000M | | 5.57 | 0.009 | 6 | 847.05 | Ψ(USUMRD100M) | 0.566 | | 0.401 | |
|  |  | |  |  |  |  | Ψ(PSUMRD100M) | -0.072 | | 0.370 | |
|  |  | |  |  |  |  | Ψ(GC1000M) | 0.339 | | 0.326 | |
|  | WC500M+PSUMRD100M+GC1000M | | 5.87 | 0.008 | 6 | 847.35 | Ψ(WC500M) | 0.494 | | 0.379 | |
|  |  | |  |  |  |  | Ψ(PSUMRD100M) | -0.442 | | 0.331 | |
|  |  | |  |  |  |  | Ψ(GC1000M) | 0.036 | | 0.341 | |
| **Gray Fox** |  | |  |  |  |  |  |  | |  | |
| FOREST COVER |  | |  |  |  |  |  |  | |  | |
|  | (.) | | 0.00 | 0.520 | 3 | 769.21 | Ψ(.) | -0.284 | | 0.297 | |
|  | FC100M | | 2.31 | 0.160 | 4 | 769.13 | Ψ(FC100M) | 0.086 | | 0.303 | |
|  | FC500M | | 2.38 | 0.160 | 4 | 769.19 | Ψ(FC500M) | -0.040 | | 0.304 | |
|  | FC1000M | | 2.38 | 0.160 | 4 | 769.20 | Ψ(FC1000M) | 0.033 | | 0.304 | |
| URBAN COVER |  | |  |  |  |  |  |  | |  | |
|  | (.) | | 0.00 | 0.440 | 3 | 769.21 | Ψ(.) | -0.284 | | 0.297 | |
|  | UC500M | | 1.35 | 0.220 | 4 | 768.17 | Ψ(UC500M) | -0.312 | | 0.310 | |
|  | UC100M | | 1.46 | 0.210 | 4 | 768.27 | Ψ(UC100M) | -0.296 | | 0.309 | |
|  | UC1000M | | 2.37 | 0.130 | 4 | 769.18 | Ψ(UC1000M) | -0.051 | | 0.302 | |
| GRASSLAND COVER | | |  |  |  |  |  |  | |  | |
|  | GC1000M | | 0.00 | 0.933 | 4 | 757.62 | Ψ(GC1000M) | 1.192 | | 0.419 | |
|  | GC500M | | 5.68 | 0.055 | 4 | 763.30 | Ψ(GC500M) | 0.792 | | 0.364 | |
|  | (.) | | 9.20 | 0.009 | 3 | 769.21 | Ψ(.) | -0.284 | | 0.297 | |
|  | GC100M | | 11.59 | 0.003 | 4 | 769.20 | Ψ(GC100M) | 0.030 | | 0.297 | |
| WATER COVER |  | |  |  |  |  |  |  | |  | |
|  | WC1000M | | 0.00 | 0.400 | 4 | 765.70 | Ψ(WC1000M) | 0.597 | | 0.347 | |
|  | (.) | | 1.12 | 0.230 | 3 | 769.21 | Ψ(.) | -0.284 | | 0.297 | |
|  | WC500M | | 1.31 | 0.210 | 4 | 767.01 | Ψ(WC500M) | 0.462 | | 0.329 | |
|  | WC100M | | 1.78 | 0.160 | 4 | 767.48 | Ψ(WC100M) | 0.415 | | 0.337 | |
| ROAD DENSITY UNPAVED | | |  |  |  |  |  |  | |  | |
|  | (.) | | 0.00 | 0.490 | 3 | 769.21 | Ψ(.) | -0.284 | | 0.297 | |
|  | USUMRD100M | | 1.59 | 0.220 | 4 | 768.41 | Ψ(USUMRD100M) | 0.277 | | 0.313 | |
|  | USUMRD1000M | | 2.38 | 0.150 | 4 | 769.20 | Ψ(USUMRD1000M) | -0.036 | | 0.301 | |
|  | USUMRD500M | | 2.39 | 0.150 | 4 | 769.21 | Ψ(USUMRD500M) | -0.025 | | 0.306 | |
| ROAD DENSITY PAVED | | |  |  |  |  |  |  | |  | |
|  | PSUMRD500M | | 0.00 | 0.410 | 4 | 764.96 | Ψ(PSUMRD500M) | -0.668 | | 0.344 | |
|  | PSUMRD1000M | | 1.03 | 0.250 | 4 | 765.98 | Ψ(PSUMRD1000M) | -0.570 | | 0.332 | |
|  | PSUMRD100M | | 1.60 | 0.180 | 4 | 766.56 | Ψ(PSUMRD100M) | -0.518 | | 0.333 | |
|  | (.) | | 1.86 | 0.160 | 3 | 769.21 | Ψ(.) | -0.284 | | 0.297 | |
| DISTANCE TO SURFACE WATER | | |  |  |  |  |  |  | |  | |
|  | (.) | | 0.00 | 0.770 | 3 | 769.21 | Ψ(.) | -0.284 | | 0.297 | |
|  | DTWATER | | 2.39 | 0.230 | 4 | 769.21 | Ψ(DTWATER) | 0.011 | | 0.301 | |
| DISTANCE TO HUMAN STRUCTURE | | |  |  |  |  |  |  | |  | |
|  | (.) | | 0.00 | 0.770 | 3 | 769.21 | Ψ(.) | -0.284 | | 0.297 | |
|  | DTSTRUC | | 2.39 | 0.230 | 4 | 769.21 | Ψ(DTSTRUC) | -0.003 | | 0.301 | |
| TOP MODELS (univariate) | | |  |  |  |  |  |  | |  | |
|  | GC1000M | | 0.00 | 0.934 | 4 | 757.62 | Ψ(GC1000M) | 1.192 | | 0.419 | |
|  | PSUMRD500M | | 7.34 | 0.024 | 4 | 764.96 | Ψ(PSUMRD500M) | -0.668 | | 0.344 | |
|  | WC1000M | | 8.08 | 0.016 | 4 | 765.70 | Ψ(WC1000M) | 0.597 | | 0.347 | |
|  | (.) | | 9.20 | 0.009 | 3 | 769.21 | Ψ(.) | -0.284 | | 0.297 | |
|  | UC500M | | 10.55 | 0.005 | 4 | 768.17 | Ψ(UC500M) | -0.312 | | 0.310 | |
|  | FC100M | | 11.51 | 0.003 | 4 | 769.13 | Ψ(FC100M) | 0.086 | | 0.303 | |
|  | USUMRD100M | | 11.58 | 0.003 | 4 | 769.20 | Ψ(USUMRD100M) | 0.277 | | 0.313 | |
|  | DTWATER | | 11.59 | 0.003 | 4 | 769.21 | Ψ(DTWATER) | 0.011 | | 0.301 | |
|  | DTSTRUC | | 11.60 | 0.003 | 4 | 769.21 | Ψ(DTSTRUC) | -0.003 | | 0.301 | |
| TOP MODELS (multivariate) | | |  |  |  |  |  |  | |  | |
|  | GC1000M+PSUMRD500M | | 0.00 | 0.503 | 5 | 755.97 | Ψ(GC1000M) | 1.087 | | 0.420 | |
|  |  | |  |  |  |  | Ψ(PSUMRD500M) | -0.470 | | 0.380 | |
|  | GC1000M+UC500M | | 1.63 | 0.223 | 5 | 757.61 | Ψ(GC1000M) | 1.182 | | 0.430 | |
|  |  | |  |  |  |  | Ψ(UC500M) | -0.034 | | 0.350 | |
|  | GC1000M+WC1000M | | 1.64 | 0.222 | 5 | 757.61 | Ψ(GC1000M) | 1.176 | | 0.475 | |
|  |  | |  |  |  |  | Ψ(WC1000M) | 0.033 | | 0.446 | |
|  | PSUMRD500M+WC1000M | | 5.27 | 0.036 | 5 | 761.25 | Ψ(PSUMRD500M) | -0.730 | | 0.374 | |
|  |  | |  |  |  |  | Ψ(WC1000M) | 0.658 | | 0.379 | |
|  | WC1000M+UC500M | | 8.15 | 0.009 | 5 | 764.12 | Ψ(WC1000M) | 0.668 | | 0.368 | |
|  |  | |  |  |  |  | Ψ(UC500M) | -0.407 | | 0.333 | |
|  | (.) | | 8.33 | 0.008 | 3 | 769.21 | Ψ(.) | -0.284 | | 0.297 | |
| ALL TOP MODELS | | |  |  |  |  |  |  | |  | |
|  | GC1000M | | 0.00 | 0.426 | 4 | 757.62 | Ψ(GC1000M) | 1.192 | | 0.419 | |
|  | GC1000M+PSUMRD500M | | 0.87 | 0.276 | 5 | 755.97 | Ψ(GC1000M) | 1.087 | | 0.420 | |
|  |  | |  |  |  |  | Ψ(PSUMRD500M) | -0.470 | | 0.380 | |
|  | GC1000M+UC500M | | 2.50 | 0.122 | 5 | 757.61 | Ψ(GC1000M) | 1.182 | | 0.430 | |
|  |  | |  |  |  |  | Ψ(UC500M) | -0.034 | | 0.350 | |
|  | GC1000M+WC1000M | | 2.51 | 0.122 | 5 | 757.61 | Ψ(GC1000M) | 1.176 | | 0.475 | |
|  |  | |  |  |  |  | Ψ(WC1000M) | 0.033 | | 0.446 | |
|  | PSUMRD500M+WC1000M | | 6.14 | 0.020 | 5 | 761.25 | Ψ(PSUMRD500M) | -0.730 | | 0.374 | |
|  |  | |  |  |  |  | Ψ(WC1000M) | 0.658 | | 0.379 | |
|  | PSUMRD500M | | 7.34 | 0.011 | 4 | 764.96 | Ψ(PSUMRD500M) | -0.668 | | 0.344 | |
|  | WC1000M | | 8.08 | 0.008 | 4 | 765.70 | Ψ(WC1000M) | 0.597 | | 0.347 | |
|  | WC1000M+UC500M | | 9.02 | 0.005 | 5 | 764.12 | Ψ(WC1000M | 0.668 | | 0.368 | |
|  |  | |  |  |  |  | Ψ(UC500M | -0.407 | | 0.333 | |
|  | (.) | | 9.20 | 0.004 | 3 | 769.21 | Ψ(.) | -0.284 | | 0.297 | |
|  | UC500M | | 10.55 | 0.002 | 4 | 768.17 | Ψ(UC500M) | -0.312 | | 0.310 | |
|  | FC100M | | 10.79 | 0.002 | 4 | 768.41 | Ψ(FC100M) | 0.086 | | 0.303 | |
|  | SUMRD1000M | | 11.58 | 0.001 | 4 | 769.19 | Ψ(SUMRD1000M) | 0.277 | | 0.313 | |
|  | DTWATER | | 11.59 | 0.001 | 4 | 769.21 | Ψ(DTWATER) | 0.011 | | 0.301 | |
|  | DTSTRUC | | 11.60 | 0.001 | 4 | 769.21 | Ψ(DTSTRUC) | -0.003 | | 0.301 | |
| **Opossum** |  | |  |  |  |  |  |  | |  | |
| FOREST COVER |  | |  |  |  |  |  |  | |  | |
|  | FC100M | | 0 | 0.9452 | 4 | 1459.87 | Ψ(FC100M) | -1.318 | | 0.396 | |
|  | FC500M | | 6.6 | 0.0348 | 4 | 1466.47 | Ψ(FC500M) | -0.916 | | 0.347 | |
|  | F1000M | | 7.95 | 0.0178 | 4 | 1467.81 | Ψ(F1000M) | -0.827 | | 0.341 | |
|  | (.) | | 12.12 | 0.0022 | 3 | 1474.38 | Ψ(.) | 0.665 | | 0.309 | |
| URBAN COVER |  | |  |  |  |  |  |  | |  | |
|  | UC100M | | 0 | 0.543 | 4 | 1464.21 | Ψ(UC100M) | 1.277 | | 0.526 | |
|  | UC500M | | 0.63 | 0.397 | 4 | 1464.83 | Ψ(UC500M) | 1.259 | | 0.548 | |
|  | UC1000M | | 4.81 | 0.049 | 4 | 1469.02 | Ψ(UC1000M) | 0.958 | | 0.551 | |
|  | (.) | | 7.78 | 0.011 | 3 | 1474.38 | Ψ(.) | 0.665 | | 0.309 | |
| GRASSLAND COVER | | |  |  |  |  |  |  | |  | |
|  | (.) | | 0 | 0.52 | 3 | 1474.38 | Ψ(.) | 0.665 | | 0.309 | |
|  | GC100M | | 2.24 | 0.17 | 4 | 1474.23 | Ψ(GC100M) | 0.126 | | 0.330 | |
|  | GC1000M | | 2.37 | 0.16 | 4 | 1474.36 | Ψ(GC1000M) | 0.046 | | 0.315 | |
|  | GC500M | | 2.38 | 0.16 | 4 | 1474.37 | Ψ(GC500M) | -0.031 | | 0.309 | |
| WATER COVER |  | |  |  |  |  |  |  | |  | |
|  | (.) | | 0 | 0.34 | 3 | 1474.38 | Ψ(.) | 0.665 | | 0.309 | |
|  | WC500M | | 0.34 | 0.29 | 4 | 1472.33 | Ψ(WC500M) | 0.522 | | 0.403 | |
|  | WC1000M | | 0.64 | 0.25 | 4 | 1472.63 | Ψ(WC1000M) | 0.471 | | 0.390 | |
|  | WC100M | | 2.08 | 0.12 | 4 | 1474.07 | Ψ(WC100M) | 0.188 | | 0.352 | |
| ROAD DENSITY UNPAVED | | |  |  |  |  |  |  | |  | |
|  | USUMRD500M | | 0 | 0.4782 | 4 | 1459.83 | Ψ(USUMRD500M) | -1.322 | | 0.406 | |
|  | USUMRD100M | | 0.16 | 0.4413 | 4 | 1459.99 | Ψ(USUMRD100M) | -1.362 | | 0.453 | |
|  | USUMRD1000M | | 3.59 | 0.0794 | 4 | 1463.42 | Ψ(USUMRD1000M) | -1.105 | | 0.381 | |
|  | (.) | | 12.16 | 0.0011 | 3 | 1474.38 | Ψ(.) | 0.665 | | 0.309 | |
| ROAD DENSITY PAVED | | |  |  |  |  |  |  | |  | |
|  | PSUMRD100M | | 0 | 0.3983 | 4 | 1464.35 | Ψ(PSUMRD100M) | 1.319 | | 0.525 | |
|  | PSUMRD500M | | 0.054 | 0.3878 | 4 | 1464.41 | Ψ(PSUMRD500M) | 1.599 | | 0.769 | |
|  | PSUMRD1000M | | 1.328 | 0.2051 | 4 | 1465.68 | Ψ(PSUMRD1000M) | 1.338 | | 0.708 | |
|  | (.) | | 7.638 | 0.0087 | 3 | 1474.38 | Ψ(.) | 0.665 | | 0.309 | |
| DISTANCE TO SURFACE WATER | | |  |  |  |  |  |  | |  | |
|  | (.) | | 0 | 0.69 | 3 | 1474.38 | Ψ(.) | 0.665 | | 0.309 | |
|  | DTWATER | | 1.56 | 0.31 | 4 | 1473.55 | Ψ(DTWATER) | -0.282 | | 0.309 | |
| DISTANCE TO HUMAN STRUCTURE | | |  |  |  |  |  |  | |  | |
|  | DTSTRUC | | 0 | 0.9982 | 4 | 1459.36 | Ψ(DTSTRUC) | -1.777 | | 0.660 | |
|  | (.) | | 12.63 | 0.0018 | 3 | 1474.38 | Ψ(.) | 0.665 | | 0.309 | |
| TOP MODELS (univariate) | | |  |  |  |  |  |  | |  | |
|  | DTSTRUC | | 0 | 0.3645 | 4 | 1459.36 | Ψ(DTSTRUC) | -1.777 | | 0.660 | |
|  | USUMRD500M | | 0.47 | 0.2886 | 4 | 1459.83 | Ψ(USUMRD500M) | -1.322 | | 0.406 | |
|  | FC100M | | 0.51 | 0.2828 | 4 | 1459.87 | Ψ(FC100M) | -1.318 | | 0.396 | |
|  | UC100M | | 4.85 | 0.0323 | 4 | 1464.21 | Ψ(UC100M) | 1.277 | | 0.526 | |
|  | PSUMRD100M | | 4.99 | 0.03 | 4 | 1464.35 | Ψ(PSUMRD100M) | 1.319 | | 0.525 | |
|  | (.) | | 12.63 | 7.00E-04 | 3 | 1474.38 | Ψ(.) | 0.665 | | 0.309 | |
|  | WC500M | | 12.97 | 6.00E-04 | 4 | 1472.33 | Ψ(WC500M) | 0.522 | | 0.403 | |
|  | DTWATER | | 14.2 | 3.00E-04 | 4 | 1473.55 | Ψ(DTWATER) | -0.282 | | 0.309 | |
|  | GC100M | | 14.87 | 2.00E-04 | 4 | 1474.23 | Ψ(GC100M) | 0.126 | | 0.330 | |
| TOP MODELS (multivariate) | | |  |  |  |  |  |  | |  | |
|  | DTSTRUC+UC100M | | 0 | 0.3777 | 5 | 1456.86 | Ψ(DTSTRUC) | -1.204 | | 0.617 | |
|  |  | |  |  |  |  | Ψ(UC100M) | 0.804 | | 0.571 | |
|  | DTSTRUC+PSUMRD100M | | 0.96 | 0.2338 | 5 | 1457.82 | Ψ(DTSTRUC) | -1.292 | | 0.679 | |
|  |  | |  |  |  |  | Ψ(PSUMRD100M) | 0.626 | | 0.542 | |
|  | USUMRD500M+UC100M | | 1.07 | 0.2215 | 5 | 1457.93 | Ψ(USUMRD500M) | -0.991 | | 0.442 | |
|  |  | |  |  |  |  | Ψ(UC100M) | 0.692 | | 0.546 | |
|  | USUMRD500M+PSUMRD100M | | 1.64 | 0.1662 | 5 | 1458.51 | Ψ(USUMRD500M) | -1.015 | | 0.465 | |
|  |  | |  |  |  |  | Ψ(PSUMRD100M) | 0.588 | | 0.547 | |
|  | (.) | | 12.62 | 7.00E-04 | 3 | 1474.38 | Ψ(.) | 0.665 | | 0.309 | |
| ALL TOP MODELS | | |  |  |  |  |  |  | |  | |
|  | DTSTRUC | | 0 | 0.1863 | 4 | 1459.36 | Ψ(DTSTRUC) | -1.777 | | 0.660 | |
|  | DTSTRUC+UC100M | | 0.015 | 0.1848 | 5 | 1456.86 | Ψ(DTSTRUC) | -1.204 | | 0.617 | |
|  |  | |  |  |  |  | Ψ(UC100M) | 0.804 | | 0.571 | |
|  | USUMRD500M | | 0.467 | 0.1475 | 4 | 1459.83 | Ψ(USUMRD500M) | -1.322 | | 0.406 | |
|  | FC100M | | 0.507 | 0.1445 | 4 | 1459.87 | Ψ(FC100M) | -1.318 | | 0.396 | |
|  | DTSTRUC+PSUMRD100M | | 0.975 | 0.1144 | 5 | 1457.82 | Ψ(DTSTRUC) | -1.292 | | 0.679 | |
|  |  | |  |  |  |  | Ψ(PSUMRD100M) | 0.626 | | 0.542 | |
|  | USUMRD500M+UC100M | | 1.083 | 0.1084 | 5 | 1457.93 | Ψ(USUMRD500M) | -0.991 | | 0.442 | |
|  |  | |  |  |  |  | Ψ(UC100M) | 0.692 | | 0.546 | |
|  | USUMRD500M+PSUMRD100M | | 1.657 | 0.0813 | 5 | 1458.51 | Ψ(USUMRD500M) | -1.015 | | 0.465 | |
|  |  | |  |  |  |  | Ψ(PSUMRD100M) | 0.588 | | 0.547 | |
|  | UC100M | | 4.848 | 0.0165 | 4 | 1464.21 | Ψ(UC100M) | 1.277 | | 0.526 | |
|  | PSUMRD100M | | 4.994 | 0.0153 | 4 | 1464.35 | Ψ(PSUMRD100M) | 1.319 | | 0.525 | |
|  | (.) | | 12.631 | 3.00E-04 | 3 | 1474.38 | Ψ(.) | 0.665 | | 0.309 | |
|  | WC500M | | 12.967 | 3.00E-04 | 4 | 1472.33 | Ψ(WC500M) | 0.522 | | 0.403 | |
|  | DTWATER | | 14.196 | 2.00E-04 | 4 | 1473.55 | Ψ(DTWATER) | -0.282 | | 0.309 | |
|  | GC100M | | 14.873 | 1.00E-04 | 4 | 1474.23 | Ψ(GC100M) | 0.126 | | 0.330 | |
| **Raccoon** |  | |  |  |  |  |  |  | |  | |
| FOREST COVER |  | |  |  |  |  |  |  | |  | |
|  | FC1000M | | 0.00 | 0.453 | 4 | 1421.68 | Ψ(FC1000M) | -0.709 | | 0.345 | |
|  | FC500M | | 0.95 | 0.282 | 4 | 1422.63 | Ψ(FC500M) | -0.625 | | 0.342 | |
|  | (.) | | 1.89 | 0.176 | 3 | 1425.96 | Ψ(.) | 0.733 | | 0.337 | |
|  | FC100M | | 3.27 | 0.088 | 4 | 1424.95 | Ψ(FC100M) | -0.354 | | 0.343 | |
| URBAN COVER |  | |  |  |  |  |  |  | |  | |
|  | UC500M | | 0.00 | 0.400 | 4 | 1422.50 | Ψ(UC500M) | 0.663 | | 0.376 | |
|  | (.) | | 1.07 | 0.230 | 3 | 1425.96 | Ψ(.) | 0.733 | | 0.337 | |
|  | UC100M | | 1.15 | 0.220 | 4 | 1423.65 | Ψ(UC100M) | 0.525 | | 0.357 | |
|  | UC1000M | | 2.01 | 0.150 | 4 | 1424.51 | Ψ(UC1000M) | 0.423 | | 0.367 | |
| GRASSLAND COVER | | |  |  |  |  |  |  | |  | |
|  | (.) | | 0.00 | 0.410 | 3 | 1425.96 | Ψ(.) | 0.733 | | 0.337 | |
|  | GC1000M | | 0.69 | 0.290 | 4 | 1424.26 | Ψ(GC1000M) | 0.465 | | 0.384 | |
|  | GC500M | | 1.78 | 0.170 | 4 | 1425.35 | Ψ(GC500M) | 0.267 | | 0.356 | |
|  | GC100M | | 2.25 | 0.130 | 4 | 1425.82 | Ψ(GC100M) | -0.118 | | 0.310 | |
| WATER COVER |  | |  |  |  |  |  |  | |  | |
|  | (.) | | 0.00 | 0.420 | 3 | 1425.96 | Ψ(.) | 0.733 | | 0.337 | |
|  | WC1000M | | 0.91 | 0.270 | 4 | 1424.47 | Ψ(WC1000M) | 0.449 | | 0.399 | |
|  | WC500M | | 1.77 | 0.170 | 4 | 1425.33 | Ψ(WC500M) | 0.280 | | 0.369 | |
|  | WC100M | | 2.17 | 0.140 | 4 | 1425.74 | Ψ(WC100M) | 0.162 | | 0.355 | |
| ROAD DENSITY UNPAVED | | |  |  |  |  |  |  | |  | |
|  | USUMRD1000M | | 0.00 | 0.760 | 4 | 1416.96 | Ψ(USUMRD1000M) | -1.036 | | 0.384 | |
|  | USUMRD500M | | 3.12 | 0.160 | 4 | 1420.08 | Ψ(USUMRD500M) | -0.828 | | 0.357 | |
|  | USUMRD100M | | 5.33 | 0.053 | 4 | 1422.29 | Ψ(USUMRD100M) | -0.699 | | 0.361 | |
|  | (.) | | 6.61 | 0.028 | 3 | 1425.96 | Ψ(.) | 0.733 | | 0.337 | |
| ROAD DENSITY PAVED | | |  |  |  |  |  |  | |  | |
|  | PSUMRD100M | | 0.00 | 0.660 | 4 | 1420.47 | Ψ(PSUMRD100M) | 0.905 | | 0.434 | |
|  | (.) | | 3.10 | 0.140 | 3 | 1425.96 | Ψ(.) | 0.733 | | 0.337 | |
|  | PSUMRD500M | | 3.18 | 0.130 | 4 | 1423.65 | Ψ(PSUMRD500M) | 0.550 | | 0.375 | |
|  | PSUMRD1000M | | 4.49 | 0.070 | 4 | 1424.96 | Ψ(PSUMRD1000M) | 0.349 | | 0.355 | |
| DISTANCE TO SURFACE WATER | | |  |  |  |  |  |  | |  | |
|  | (.) | | 0.00 | 0.660 | 3 | 1425.96 | Ψ(.) | 0.733 | | 0.337 | |
|  | DTWATER | | 1.28 | 0.340 | 4 | 1424.85 | Ψ(DTWATER) | -0.347 | | 0.327 | |
| DISTANCE TO HUMAN STRUCTURE | | |  |  |  |  |  |  | |  | |
|  | DTSTRUC | | 0.00 | 0.830 | 4 | 1420.44 | Ψ(DTSTRUC) | -0.615 | | 0.268 | |
|  | (.) | | 3.13 | 0.170 | 3 | 1425.96 | Ψ(.) | 0.733 | | 0.337 | |
| TOP MODELS (univariate) | | |  |  |  |  |  |  | |  | |
|  | DTSTRUC | | 0.00 | 0.707 | 4 | 1414.47 | Ψ(DTSTRUC) | -0.615 | | 0.268 | |
|  | USUMRD1000M | | 2.48 | 0.204 | 4 | 1416.96 | Ψ(USUMRD1000M) | -1.036 | | 0.384 | |
|  | PSUMRD100M | | 6.00 | 0.035 | 4 | 1420.47 | Ψ(PSUMRD100M) | 0.905 | | 0.434 | |
|  | FC1000M | | 7.21 | 0.019 | 4 | 1421.68 | Ψ(FC1000M) | -0.709 | | 0.345 | |
|  | UC500M | | 8.03 | 0.013 | 4 | 1422.50 | Ψ(UC500M) | 0.663 | | 0.376 | |
|  | (.) | | 9.09 | 0.008 | 3 | 1425.96 | Ψ(.) | 0.733 | | 0.337 | |
|  | GC1000M | | 9.78 | 0.005 | 4 | 1424.26 | Ψ(GC1000M) | 0.465 | | 0.384 | |
|  | WC1000M | | 10.00 | 0.005 | 4 | 1424.47 | Ψ(WC1000M) | 0.449 | | 0.399 | |
|  | DTWATER | | 10.37 | 0.004 | 4 | 1424.85 | Ψ(DTWATER) | -0.347 | | 0.327 | |
| TOP MODELS (multivariate) | | |  |  |  |  |  |  | |  | |
|  | DTSTRUC+PSUMRD100M | | 0.00 | 0.312 | 5 | 1414.02 | Ψ(DTSTRUC) | -1.318 | | 0.688 | |
|  |  | |  |  |  |  | Ψ(PSUMRD100M) | 0.311 | | 0.469 | |
|  | DTSTRUC+UC500M | | 0.42 | 0.253 | 5 | 1414.44 | Ψ(DTSTRUC) | -1.494 | | 0.734 | |
|  |  | |  |  |  |  | Ψ(UC500M) | 0.088 | | 0.445 | |
|  | DTSTRUC+FC1000M | | 0.45 | 0.250 | 5 | 1414.46 | Ψ(DTSTRUC) | -1.516 | | 0.841 | |
|  |  | |  |  |  |  | Ψ(FC1000M) | -0.051 | | 0.510 | |
|  | USUMRD1000M+PSUMRD100M | | 2.27 | 0.100 | 5 | 1416.29 | Ψ(USUMRD1000M) | -0.835 | | 0.441 | |
|  |  | |  |  |  |  | Ψ(PSUMRD100M) | 0.377 | | 0.474 | |
|  | USUMRD1000M+UC500M | | 2.84 | 0.075 | 5 | 1416.86 | Ψ(USUMRD1000M) | -0.959 | | 0.448 | |
|  |  | |  |  |  |  | Ψ(UC500M) | 0.138 | | 0.439 | |
|  | (.) | | 7.04 | 0.009 | 3 | 1425.96 | Ψ(.) | 0.733 | | 0.337 | |
| ALL TOP MODELS | | |  |  |  |  |  |  | |  | |
|  | DTSTRUC | | 0.00 | 0.392 | 4 | 1414.47 | Ψ(DTSTRUC) | -0.615 | | 0.268 | |
|  | DTSTRUC+PSUMRD100M | | 2.05 | 0.140 | 5 | 1414.02 | Ψ(DTSTRUC) | -1.318 | | 0.688 | |
|  |  | |  |  |  |  | Ψ(PSUMRD100M) | 0.311 | | 0.469 | |
|  | DTSTRUC+UC500M | | 2.47 | 0.114 | 5 | 1414.44 | Ψ(DTSTRUC) | -1.494 | | 0.734 | |
|  |  | |  |  |  |  | Ψ(UC500M) | 0.088 | | 0.445 | |
|  | USUMRD1000M | | 2.48 | 0.113 | 4 | 1416.96 | Ψ(USUMRD1000M) | -1.036 | | 0.384 | |
|  | DTSTRUC+FC1000M | | 2.50 | 0.112 | 5 | 1414.46 | Ψ(DTSTRUC) | -1.516 | | 0.841 | |
|  |  | |  |  |  |  | Ψ(FC1000M) | -0.051 | | 0.510 | |
|  | USUMRD1000M+PSUMRD100M | | 4.32 | 0.045 | 5 | 1416.29 | Ψ(USUMRD1000M) | -0.835 | | 0.441 | |
|  |  | |  |  |  |  | Ψ(PSUMRD100M) | 0.377 | | 0.474 | |
|  | USUMRD1000M+UC500M | | 4.90 | 0.034 | 5 | 1416.86 | Ψ(USUMRD1000M) | -0.959 | | 0.448 | |
|  |  | |  |  |  |  | Ψ(UC500M) | 0.138 | | 0.439 | |
|  | PSUMRD100M | | 6.00 | 0.020 | 4 | 1420.47 | Ψ(PSUMRD100M) | 0.905 | | 0.434 | |
|  | FC1000M | | 7.21 | 0.011 | 4 | 1421.68 | Ψ(FC1000M) | -0.709 | | 0.345 | |
|  | UC500M | | 8.03 | 0.007 | 4 | 1422.50 | Ψ(UC500M) | 0.663 | | 0.376 | |
|  | (.) | | 9.09 | 0.004 | 3 | 1425.96 | Ψ(.) | 0.733 | | 0.337 | |
|  | GC1000M | | 9.78 | 0.003 | 4 | 1424.26 | Ψ(GC1000M) | 0.465 | | 0.384 | |
|  | WC1000M | | 10.00 | 0.003 | 4 | 1424.47 | Ψ(WC1000M) | 0.449 | | 0.399 | |
|  | DTWATER | | 10.37 | 0.002 | 4 | 1424.85 | Ψ(DTWATER) | -0.347 | | 0.327 | |
| **Striped Skunk** |  | |  |  |  |  |  |  | |  | |
| FOREST COVER |  | |  |  |  |  |  |  | |  | |
|  | FC500M | | 0.00 | 0.404 | 4 | 708.01 | Ψ(FC500M) | 0.875 | | 0.337 | |
|  | FC1000M | | 0.11 | 0.382 | 4 | 708.12 | Ψ(FC1000M) | 0.872 | | 0.339 | |
|  | FC100M | | 1.55 | 0.185 | 4 | 709.57 | Ψ(FC100M) | 0.769 | | 0.326 | |
|  | (.) | | 5.25 | 0.029 | 3 | 715.65 | Ψ(.) | -0.380 | | 0.298 | |
| URBAN COVER |  | |  |  |  |  |  |  | |  | |
|  | UC1000M | | 0.00 | 0.490 | 4 | 710.07 | Ψ(UC1000M) | -0.824 | | 0.393 | |
|  | UC500M | | 1.65 | 0.220 | 4 | 711.72 | Ψ(UC500M) | -0.636 | | 0.338 | |
|  | UC100M | | 1.86 | 0.190 | 4 | 711.93 | Ψ(UC100M) | -0.613 | | 0.333 | |
|  | (.) | | 3.19 | 0.100 | 3 | 715.65 | Ψ(.) | -0.380 | | 0.298 | |
| GRASSLAND COVER | | |  |  |  |  |  |  | |  | |
|  | (.) | | 0.00 | 0.510 | 3 | 715.65 | Ψ(.) | -0.380 | | 0.298 | |
|  | GC1000M | | 2.12 | 0.180 | 4 | 715.38 | Ψ(GC1000M) | 0.156 | | 0.298 | |
|  | GC500M | | 2.38 | 0.160 | 4 | 715.63 | Ψ(GC500M) | -0.042 | | 0.303 | |
|  | GC100M | | 2.39 | 0.150 | 4 | 715.65 | Ψ(GC100M) | -0.022 | | 0.302 | |
| WATER COVER |  | |  |  |  |  |  |  | |  | |
|  | (.) | | 0.00 | 0.430 | 3 | 715.65 | Ψ(.) | -0.380 | | 0.298 | |
|  | WC100M | | 1.07 | 0.250 | 4 | 714.33 | Ψ(WC100M) | -0.402 | | 0.388 | |
|  | WC500M | | 1.86 | 0.170 | 4 | 715.12 | Ψ(WC500M) | -0.229 | | 0.323 | |
|  | WC1000M | | 2.22 | 0.140 | 4 | 715.48 | Ψ(WC1000M) | 0.126 | | 0.298 | |
| ROAD DENSITY UNPAVED | | |  |  |  |  |  |  | |  | |
|  | (.) | | 0.00 | 0.380 | 3 | 715.65 | Ψ(.) | -0.380 | | 0.298 | |
|  | USUMRD100M | | 0.33 | 0.320 | 4 | 713.59 | Ψ(USUMRD100M) | 0.437 | | 0.313 | |
|  | USUMRD500M | | 1.41 | 0.190 | 4 | 714.67 | Ψ(USUMRD500M) | 0.297 | | 0.301 | |
|  | USUMRD1000M | | 2.30 | 0.120 | 4 | 715.56 | Ψ(USUMRD1000M) | 0.093 | | 0.299 | |
| ROAD DENSITY PAVED | | |  |  |  |  |  |  | |  | |
|  | PSUMRD100M | | 0.00 | 0.388 | 4 | 709.85 | Ψ(PSUMRD100M) | -0.825 | | 0.380 | |
|  | PSUMRD500M | | 0.44 | 0.311 | 4 | 710.29 | Ψ(PSUMRD500M) | -0.768 | | 0.358 | |
|  | PSUMRD1000M | | 1.04 | 0.230 | 4 | 710.89 | Ψ(PSUMRD1000M) | -0.718 | | 0.353 | |
|  | (.) | | 3.41 | 0.071 | 3 | 715.65 | Ψ(.) | -0.380 | | 0.298 | |
| DISTANCE TO SURFACE WATER | | |  |  |  |  |  |  | |  | |
|  | (.) | | 0.00 | 0.760 | 3 | 715.65 | Ψ(.) | -0.380 | | 0.298 | |
|  | DTWATER | | 2.30 | 0.240 | 4 | 715.56 | Ψ(DTWATER) | 0.092 | | 0.302 | |
| DISTANCE TO HUMAN STRUCTURE | | |  |  |  |  |  |  | |  | |
|  | (.) | | 0.00 | 0.770 | 3 | 715.65 | Ψ(.) | -0.380 | | 0.298 | |
|  | DTSTRUC | | 2.37 | 0.230 | 4 | 715.62 | Ψ(DTSTRUC) | 0.051 | | 0.300 | |
| TOP MODS (univariate) | | |  |  |  |  |  |  | |  | |
|  | FC500M | | 0.00 | 0.498 | 4 | 708.01 | Ψ(FC500M) | 0.875 | | 0.337 | |
|  | PSUMRD100M | | 1.84 | 0.199 | 4 | 709.85 | Ψ(PSUMRD100M) | -0.825 | | 0.380 | |
|  | UC1000M | | 2.06 | 0.178 | 4 | 710.07 | Ψ(UC1000M) | -0.824 | | 0.393 | |
|  | (.) | | 5.25 | 0.036 | 3 | 715.65 | Ψ(.) | -0.380 | | 0.298 | |
|  | USUMRD100M | | 5.58 | 0.031 | 4 | 713.59 | Ψ(USUMRD100M) | 0.437 | | 0.313 | |
|  | WC100M | | 6.32 | 0.021 | 4 | 714.33 | Ψ(WC100M) | -0.402 | | 0.388 | |
|  | GC1000M | | 7.37 | 0.013 | 4 | 715.38 | Ψ(GC1000M) | 0.156 | | 0.298 | |
|  | DTWATER | | 7.55 | 0.011 | 4 | 715.56 | Ψ(DTWATER) | 0.092 | | 0.302 | |
|  | DTSTRUC | | 7.61 | 0.011 | 4 | 715.62 | Ψ(DTSTRUC) | 0.051 | | 0.300 | |
| TOP MODS (multivariate) | | |  |  |  |  |  |  | |  | |
|  | FC500M+USUMRD100M | | 0.00 | 0.339 | 5 | 707.97 | Ψ(FC500M) | 0.923 | | 0.411 | |
|  |  | |  |  |  |  | Ψ(USUMRD100M) | -0.080 | | 0.390 | |
|  | PSUMRD100M+UC1000M | | 0.29 | 0.292 | 5 | 708.26 | Ψ(PSUMRD100M) | -0.546 | | 0.424 | |
|  |  | |  |  |  |  | Ψ(UC1000M) | -0.523 | | 0.434 | |
|  | UC1000M+USUMRD100M | | 1.74 | 0.142 | 5 | 709.71 | Ψ(UC1000M) | -0.742 | | 0.413 | |
|  |  | |  |  |  |  | Ψ(USUMRD100M) | 0.195 | | 0.330 | |
|  | PSUMRD100M+USUMRD100M | | 1.77 | 0.140 | 5 | 709.74 | Ψ(PSUMRD100M) | -0.761 | | 0.421 | |
|  |  | |  |  |  |  | Ψ(USUMRD100M) | 0.115 | | 0.348 | |
|  | (.) | | 2.78 | 0.084 | 3 | 715.65 | Ψ(.) | -0.380 | | 0.298 | |
| ALL TOP MODS |  | |  |  |  |  |  |  | |  | |
|  | FC500M | | 0.00 | 0.358 | 4 | 708.01 | Ψ(FC500M) | 0.875 | | 0.337 | |
|  | PSUMRD100M | | 1.84 | 0.143 | 4 | 709.85 | Ψ(PSUMRD100M) | -0.825 | | 0.380 | |
|  | UC1000M | | 2.06 | 0.128 | 4 | 710.07 | Ψ(UC1000M) | -0.824 | | 0.393 | |
|  | FC500M+USUMRD100M | | 2.47 | 0.104 | 5 | 707.97 | Ψ(FC500M) | 0.923 | | 0.411 | |
|  |  | |  |  |  |  | Ψ(USUMRD100M) | -0.080 | | 0.390 | |
|  | PSUMRD100M+UC1000M | | 2.76 | 0.090 | 5 | 708.26 | Ψ(PSUMRD100M) | -0.546 | | 0.424 | |
|  |  | |  |  |  |  | Ψ(UC1000M) | -0.523 | | 0.434 | |
|  | UC1000M+USUMRD100M | | 4.21 | 0.044 | 5 | 709.71 | Ψ(UC1000M) | -0.742 | | 0.413 | |
|  |  | |  |  |  |  | Ψ(USUMRD100M) | 0.195 | | 0.330 | |
|  | PSUMRD100M+USUMRD100M | | 4.24 | 0.043 | 5 | 709.74 | Ψ(PSUMRD100M) | -0.761 | | 0.421 | |
|  |  | |  |  |  |  | Ψ(USUMRD100M) | 0.115 | | 0.348 | |
|  | (.) | | 5.25 | 0.026 | 3 | 715.65 | Ψ(.) | -0.380 | | 0.298 | |
|  | USUMRD100M | | 5.58 | 0.022 | 4 | 713.59 | Ψ(USUMRD100M) | 0.437 | | 0.313 | |
|  | WC100M | | 6.32 | 0.015 | 4 | 714.33 | Ψ(WC100M) | -0.402 | | 0.388 | |
|  | GC1000M | | 7.37 | 0.009 | 4 | 715.38 | Ψ(GC1000M) | 0.156 | | 0.298 | |
|  | DTWATER | | 7.55 | 0.008 | 4 | 715.56 | Ψ(DTWATER) | 0.092 | | 0.302 | |
|  | DTSTRUC | | 7.61 | 0.008 | 4 | 715.62 | Ψ(DTSTRUC) | 0.051 | | 0.300 | |
| ^a^ FC100M, percent forest cover within 100m radius; FC500M, percent forest cover within 500m radius; FC1000M, percent forest cover within 1000m radius; UC100M, percent urban cover within 100m radius; UC500M, percent urban cover within 500m radius; UC1000M, percent urban cover within 1000m radius; GC100M, percent grassland cover within 100m radius; GC500M, percent grassland cover within 500m radius; GC1000M, percent grassland cover within 1000m radius; WC100M, percent water cover within 100m radius; WC500M, percent water cover within 500m radius; WC1000M, percent water cover within 1000m radius; USUMRD100M, summed length of unpaved roads in 100m radius; USUMRD500M, summed length of unpaved roads in 500m radius; USUMRD1000M, summed length of unpaved roads in 1000m radius; PSUMRD100M, summed length of paved roads in 100m radius; PSUMRD500M, summed length of paved roads in 500m radius; PSUMRD1000M, summed length of paved roads in 1000m radius; DTWATER, distance to nearest surface water; DTSTRUC, distance to nearest human structure; (.), null model. | | | | | | | | | | | |
| ^b^ Akaike’s Information Criterion. | | | | | | | | | | | |
| ^c^ Model weight. | | | | | | | | | | | |
| ^d^ Number of model parameters. | | | | | | | | | | | |
| ^e^ Difference in -2Log(Likelihood) of the current model and -2log(Likelihood) of the saturated model as a measure of model fit. | | | | | | | | | | | |
| ^f^ Estimate of effect size. | | | | | | | | | | | |
| ^g^ Standard error of effect size. | | | | | | | | | | | |
